# Dual Mo‐Doping in BiVO_4_/FeCoNiO_x_ Photoanode Enables Near‐Theoretical Photocurrent Density via Synergistic Bulk‐Surface Engineering for Solar Water Splitting

**DOI:** 10.1002/advs.202509037

**Published:** 2025-07-16

**Authors:** Rongzhe Zhao, Yuchen Zhou, Peng Guo, Rong Mo, Yonghua Tang, Hongxing Li

**Affiliations:** ^1^ Hunan Key Laboratory for Micro‐Nano Energy Materials and Devices School of Physics and Optoelectronics Xiangtan University Xiangtan Hunan 411105 P. R. China

**Keywords:** bismuth vanadate, charge transfer, Mo doping, Oxygen evolution cocatalyst, photoelectrochemical water splitting

## Abstract

Bismuth vanadate (BiVO₄) is an auspicious photoanode material for photoelectrochemical (PEC) water splitting, but its performance is fundamentally limited by severe charge recombination and sluggish kinetics of the oxygen evolution reaction (OER). Herein, a dual electronic modulation strategy is developed by incorporating molybdenum (Mo) dopants simultaneously into the FeCoNiO_x_ cocatalyst surface and the bulk phase of BiVO₄. The resulting Mo:FeCoNiO_x_/Mo:BiVO₄ photoanode delivers a near‐theoretical photocurrent density of 7.15 mA cm⁻^2^ at 1.23 V versus reversible hydrogen electrode (RHE) under AM 1.5 G illumination. This exceptional performance arises from the Mo‐triggered cross‐scale electronic reconstruction: (1) In the bulk, Mo substitution at vanadium (V) sites in BiVO₄ enhances charge transport via n‐type doping; (2) At the surface, Mo incorporation into FeCoNiO_x_ triggers electron redistribution, creating localized electron reservoirs at Fe/Co/Ni sites. Combined density functional theory (DFT) calculations and experimental validation reveal that the reconfigured Fe sites serve a dual function as efficient hole traps and highly active OER centers, reducing the reaction energy barrier (ΔG_*OH_) by 1.26 eV. Moreover, the optimized interfacial charge transport boosts carrier separation efficiency from 84.9% to 96.5% and accelerates hole migration by 2.7‐fold compared to pristine BiVO₄. This work provides insights into multi‐scale electronic engineering for solar energy conversion.

## Graphical abstract

This work highlights a dual‐electron modulation strategy by incorporating molybdenum (Mo) dopants simultaneously into the surface of the FeCoNiO_x_ cocatalyst and the bulk phase of the BiVO₄ semiconductor. The Mo‐triggered cross‐scale electronic reconstruction synergistically enhances charge transport dynamics and water oxidation kinetics, enabling the optimized Mo:FeCoNiO_x_/Mo:BiVO₄ photoanode achieves a near‐theoretical photocurrent density of 7.15 mA cm⁻^2^ at 1.23 V versus reversible hydrogen electrode (RHE) under AM 1.5 G illumination.

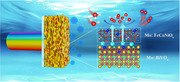

## 1 Introduction

Semiconductor‐based photoelectrochemical (PEC) water‐splitting technology, which directly converts intermittent solar energy into green hydrogen, has emerged as a promising solution to alleviate the global energy crisis.^[^ ^1^, ^2^, ^3^ ^]^ However, constructing efficient overall water‐splitting systems faces significant challenges due to sluggish water oxidation reaction kinetics involving uphill energy barriers and multi‐electron transfer processes at photoanodes.^[^ ^4^ ^]^ Among existing photoanode materials, bismuth vanadate (BiVO₄) stands out as one of the most promising candidates for solar water splitting owing to its suitable bandgap (2.4 eV) and appropriate band edge positions.^[^ ^5^, ^6^, ^7^ ^]^ Nevertheless, pure BiVO₄ photoanodes suffer from severe charge recombination and slow water oxidation kinetics caused by poor charge transport properties and short hole diffusion lengths (<70 nm), resulting in PEC performance far below the theoretical maximum (7.5 mA cm⁻^2^ under AM 1.5 G illumination).^[^ ^8^, ^9^ ^]^ Over recent decades, various strategies, including heteroatom doping,^[^ ^10^, ^11^, ^12^ ^]^ structural modulation,^[^ ^13^, ^14^ ^]^ crystal facet engineering,^[^ ^15^, ^16^ ^]^ heterojunction construction,^[^ ^17^, ^18^ ^]^ defect engineering,^[^ ^19^, ^20^ ^]^ and loading of oxygen evolution cocatalysts (OECs),^[^ ^21^, ^22^, ^23^ ^]^ have been developed to overcome these limitations and enhance the PEC water oxidation activity of BiVO₄ photoanodes. Surface modification of BiVO₄ with OECs has become one of the most favored approaches.^[^ ^24^, ^25^ ^]^ For instance, BiVO₄ photoanodes modified with FeOOH/NiOOH dual OECs achieved a photocurrent density of 4.5 mA cm⁻^2^ at 1.23 V versus reversible hydrogen electrode (RHE).^[^ ^26^ ^]^ Although the performance enhancement mechanisms of OECs have been extensively investigated, the relationship between electronic structure optimization and catalytic efficiency in multi‐metallic cocatalyst systems remains systematically underexplored.^[^ ^27^, ^28^ ^]^ For example, Zhang et al. reported a nitrogen doping strategy to rationally regulate the electronic structure of NiFeO_x_ cocatalysts, enabling the N:NiFeO_x_/BiVO₄ photoanode to achieve a high photocurrent density of up to 6.4 mA cm⁻^2^ at 1.23 V_RHE_.^[^ ^29^ ^]^ This confirmed the effectiveness of electronic engineering strategies.

On the other hand, it should be noted that the oxygen evolution reaction (OER) involves a complex four‐electron transfer process whose efficiency depends not only on the intrinsic activity of OECs but also critically on the charge transfer dynamics at the photoanode/cocatalyst interface.^[^ ^30^, ^31^ ^]^ Although surface modification with highly active OECs (e.g., Fe/NiOOH, Fe/Co/NiO_x_) can partially suppress charge recombination, the limited driving force at the OEC/BiVO₄ interface often hinders efficient extraction of photogenerated holes.^[^ ^32^ ^]^ To address this bottleneck, researchers have proposed introducing hole transport layers (HTLs) at the interface to enhance charge separation efficiency.^[^ ^33^, ^34^ ^]^ For example, Park et al. employed black phosphorus (BP) nanosheets as a photogenerated hole extraction layer to facilitate hole transfer from BiVO₄ to OEC layers (NiOOH, MnO_x_, and CoOOH), thereby improving charge separation and transfer efficiency in PEC water splitting.^[^ ^35^ ^]^ However, introducing such third‐phase components often creates additional interfacial resistance and limits performance improvement. Therefore, without introducing a further phase, precisely regulating the electronic structure of OECs and their interfacial coupling strength with BiVO₄ to simultaneously suppress interfacial charge recombination and optimize hole transport kinetics is expected further to enhance the PEC water splitting performance of BiVO₄ systems.

Herein, we successfully achieved dual electronic modulation in the photoelectrode system by incorporating molybdenum (Mo) atoms into the surface of the FeCoNiO_x_ cocatalyst and the bulk phase of the BiVO₄ semiconductor. The optimized Mo:FeCoNiO_x_/Mo:BiVO₄ photoanode demonstrated exceptional performance, delivering a photocurrent density of 7.15 mA cm⁻^2^ under AM 1.5 G irradiation (100 mW cm⁻^2^) at 1.23 V_RHE_, approaching its theoretical limit (7.5 mA cm⁻^2^), while simultaneously enhancing operational stability. This breakthrough performance stems from the cross‐scale electronic reconstruction induced by Mo doping: At the bulk level, Mo preferentially occupies vanadium (V) sites in the BiVO₄ lattice to form n‐type doping channels, while at the surface level, substitution of nickel (Ni) sites in FeCoNiO_x_ by Mo triggers electron redistribution. Through synergistic integration of density functional theory (DFT) calculations and experimental characterization, we systematically elucidated the multifunctional mechanisms of Mo: 1) In the FeCoNiO_x_ cocatalyst, Mo doping induces electron enrichment at Fe/Co/Ni sites, creating localized electron reservoirs; 2) The reconfigured iron (Fe) active sites exhibit dual functionality as both hole‐trapping centers and highly active OER sites, significantly reducing the reaction energy barrier (ΔG_*OH_ decreased by 1.26 eV); 3) Optimized interfacial charge transport pathways enable a breakthrough enhancement in carrier separation efficiency from 84.9% to 96.5%, accompanied by a 2.7‐fold improvement in hole migration rate compared to pristine BiVO₄. This work deciphers the intrinsic synergy between OER catalyst electronic configuration and photocatalyst engineering, establishing a “bulk doping‐surface modification‐interface transport” trinity design paradigm that provides novel theoretical insights and experimental methodologies for developing high‐efficiency photoelectrocatalytic systems.

## 2 Results and Discussion

### 2.1 Properties Synthesis and Characterization

**Figure 1:**
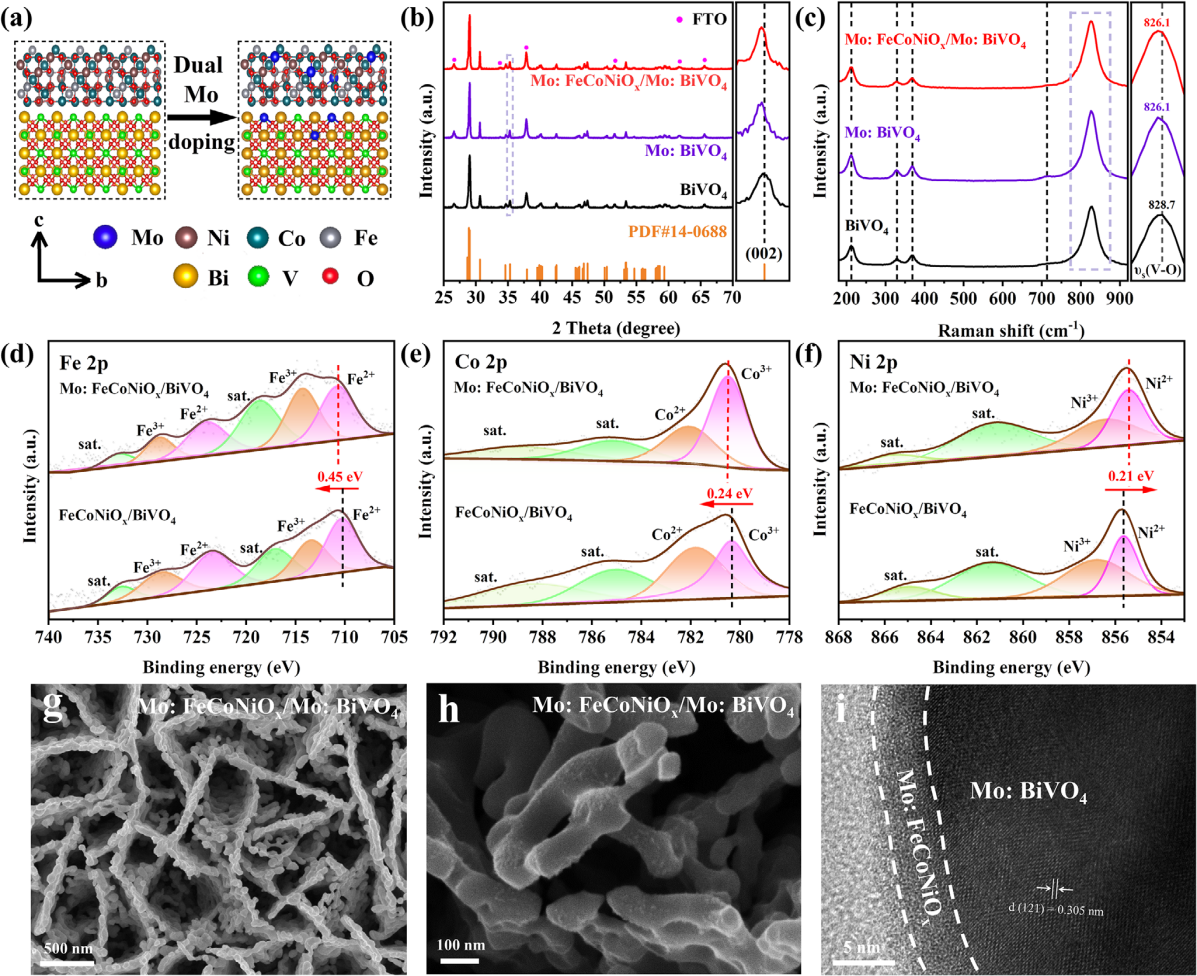
a) Schematic illustration of Mo:FeCoNiO_x_/Mo:BiVO₄. b) XRD patterns and c) Raman spectra of BiVO_4_, Mo:BiVO₄, and Mo:FeCoNiO_x_/Mo:BiVO₄ photoanodes. d) Fe 2p, e) Ni 2p, and f) Co 2p spectra of FeCoNiO_x_/BiVO₄ and Mo:FeCoNiO_x_/BiVO₄ photoanodes. g), h) SEM images of Mo:FeCoNiO_x_/Mo:BiVO₄ photoanode. i) HRTEM image of Mo:FeCoNiO_x_/Mo:BiVO₄ photoanode.

The Molybdenum (Mo)‐doped Mo:FeCoNiO_x_/Mo:BiVO₄ were prepared through a photo‐assisted electrodeposition protocol (Figure S1, Supporting Information). Mo is expected to be a substitute for nickel (Ni) sites in the surface cocatalyst and vanadium (V) sites in the photocatalyst. This inference has been validated through multiple characterization techniques and theoretical calculations (**Figure** **1**a). X‐ray diffraction (XRD) analysis confirmed the crystalline structure of the fabricated photoanodes (Figure 1b). All samples exhibited characteristic diffraction peaks corresponding to BiVO₄ (JCPDS 14–0688). The nearly identical XRD patterns of BiVO₄ hitherto and Mo:BiVO₄ hitherto indicate that Mo⁶⁺ doping did not alter the host phase. However, high‐resolution analysis (Figures S2b and S3b, Supporting Information) revealed a subtle 0.1° shift toward lower 2θ values for the (002) diffraction peak at ∼35.3° in Mo:BiVO₄, consistent with lattice expansion caused by partial substitution of smaller V⁵⁺ ions (0.36 Å) with larger Mo⁶⁺ ions (0.41 Å).^[^ ^36^ ^]^ Crucially, no peak shifts or new crystalline phases emerged after Mo:FeCoNiO_x_ deposition, confirming the amorphous nature of the oxygen evolution cocatalyst (OEC). Raman spectroscopy further elucidated structural modifications (Figure 1c). All samples displayed characteristic monoclinic BiVO₄ vibrational modes at 210 cm⁻¹ (external mode), 324 cm⁻¹ (δ_as_(VO₄^3^⁻)), 366 cm⁻¹ (δ_s_(VO₄^3^⁻)), 712 cm⁻¹ (ν_as_(V–O)), and 830 cm⁻¹ (ν_s_(V–O)).^[^ ^37^ ^]^ High‐resolution analysis (Figure S4c, Supporting Information) revealed a 2.6 cm⁻¹ redshift in the symmetric V–O stretching mode (828.7 → 826.1 cm⁻¹) for Mo:BiVO₄, providing direct evidence of Mo incorporation.^[^ ^38^, ^39^ ^]^ Morphological characterization revealed that the worm‐like porous architecture (100–200 nm) of BiVO₄ remained intact after Mo doping (Figure S5a,b, Supporting Information), with Mo:FeCoNiO_x_ deposition introducing surface nanoparticles that enhanced electrolyte contact (Figure 1g,h). Cross‐sectional scanning electron microscopy (SEM) image reveals that the Mo:FeCoNiO_x_/Mo:BiVO₄ photoanode exhibits a thickness of ≈2 µm (Figure S6, Supporting Information). High‐resolution transmission electron microscopy (HRTEM) analysis (Figure 1i) further confirmed a conformal 3–5 nm amorphous Mo:FeCoNiO_x_ coating on crystalline BiVO₄ ((121) planes, d_(121)_ = 0.305 nm). Energy dispersive spectroscopy (EDS) elemental mapping images presented in Figure S7 (Supporting Information) show that all elements are uniformly distributed in the Mo:FeCoNiO_x_/Mo:BiVO₄, completing the structural characterization of this hierarchically modified photoanode system. These results confirm that the Mo double‐doped cocatalyst deposition retains the crystalline BiVO₄ structure while forming the amorphous OEC layer.

X‐ray photoelectron spectroscopy (XPS) analysis was systematically employed to validate the incorporation of Mo into the FeCoNiO_x_ cocatalyst and elucidate its electronic effects. To eliminate interference from the Mo:BiVO₄ substrate, undoped BiVO₄ was used as the support for XPS characterization. The Mo 3d spectrum (Figure S8, Supporting Information) exhibited characteristic doublet peaks at 232.0 eV (3d_5/2_) and 235.2 eV (3d_3/2_), unambiguously confirming the presence of Mo⁶⁺ species in the cocatalyst.^[^ ^40^ ^]^ Detailed analysis of the transition metal oxidation states revealed: (i) The Fe 2p spectrum (Figure 1d) displayed primary peaks at 710.8 eV (2p_3/2_) and 723.3 eV (2p_1/2_), accompanied by satellite features at 717.1 and 723.6 eV, characteristic of iron oxides.^[^ ^41^ ^]^ Peak deconvolution identified mixed Fe^2^⁺ (710.3 eV) and Fe^3^⁺ (713.4 eV) states. (ii) Co 2p₃_/_₂ and Ni 2p₃_/_₂ spectra (Figure 1e,f) indicated the coexistence of M^2^⁺ (Co: 782.1 eV; Ni: 855.6 eV) and M^3^⁺ (Co: 780.3 eV; Ni: 856.8 eV) species.^[^ ^42^, ^43^ ^]^ Notably, the comparative analysis demonstrated that the Fe 2p and Co 2p peaks of Mo:FeCoNiO_x_/BiVO₄ shifted to higher binding energies by 0.45 and 0.24 eV, respectively, relative to FeCoNiO_x_/BiVO₄, while Ni 2p exhibited a negative shift. These shifts are attributed to the higher electronegativity of Mo⁶⁺.^[^ ^44^ ^]^ Furthermore, the relative proportions of Fe^3^⁺ and Co^3^⁺ species in Mo:FeCoNiO_x_/BiVO₄ increased significantly. Based on these findings, it can be inferred that the partial substitution of Ni sites by Mo atoms in the FeCoNiO_x_ cocatalyst enriches the electron density at the Fe, Co, and Ni sites. In addition, electron localization function analysis (Figure S9, Supporting Information) revealed that Mo doping significantly enhances electron enrichment at the Fe/Co/Ni sites in Mo:FeCoNiO_x_ compared to FeCoNiO_x_. Furthermore, the electron‐rich Fe sites efficiently capture photogenerated holes generated on the BiVO₄ surface, significantly enhancing the oxygen evolution reaction (OER) activity. Consequently, Mo doping in the FeCoNiO_x_ cocatalyst effectively improves the OER activity and stability of the BiVO₄ photoanode.

### 2.2 Photoelectrochemical Performances

**Figure 2:**
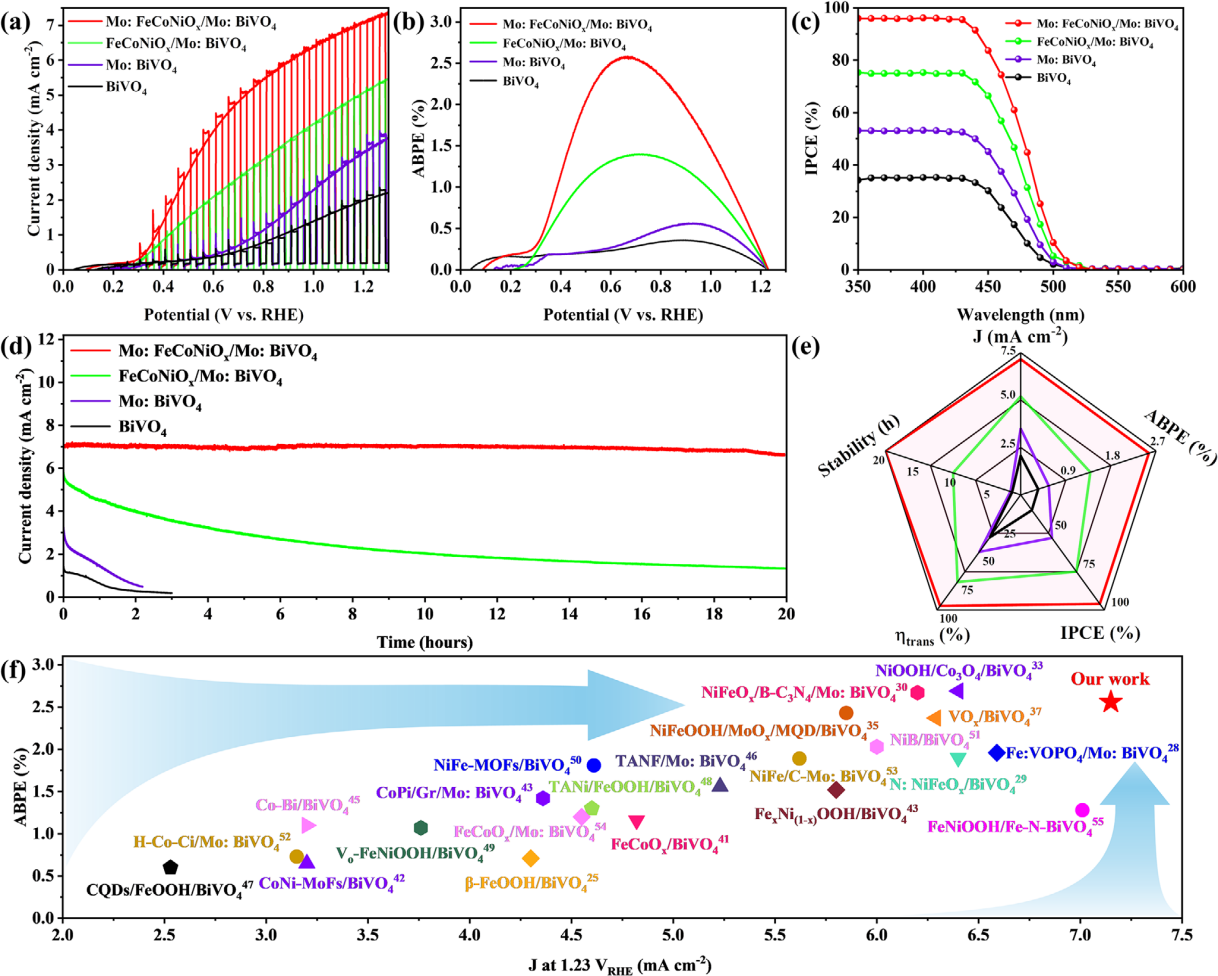
a) J‐V curves, b) ABPE curves, c) IPCE curves obtained at 1.23 V_RHE_, and d) Photocurrent density versus time curves at 1.23 V_RHE_ of BiVO₄, Mo:BiVO₄, FeCoNiO_x_/Mo:BiVO₄ and Mo:FeCoNiO_x_/Mo:BiVO₄ photoanodes in 0.25 mol L^−1^ K₃BO₃ electrolyte (pH = 9.5) under AM 1.5G illumination (100 mW cm⁻^2^). e) PEC performance comparison of the obtained photoanodes. f) PEC performance comparison of recently reported BiVO₄‐based photoanodes.

The photoelectrochemical (PEC) water oxidation performance of the fabricated photoanodes was evaluated in a 0.25 m K₃BO₃ electrolyte (pH 9.5) using a standard three‐electrode system under backside AM 1.5G illumination (100 mW cm⁻^2^). As depicted in the current density‐voltage (J‐V) curves (**Figure** **2**a), the pristine BiVO₄ photoanode exhibited a modest photocurrent density of 2.06 mA cm⁻^2^ at 1.23 V_RHE_. In contrast, Mo:BiVO₄ demonstrated a significantly enhanced photocurrent density of 3.49 mA cm⁻^2^ under identical conditions. The PEC activity was further amplified by depositing the FeCoNiO_x_ cocatalyst on Mo:BiVO₄, achieving a photocurrent density of 5.19 mA cm⁻^2^ at 1.23 V_RHE_, a 2.5‐fold improvement over pristine BiVO₄. Remarkably, the dual‐modified Mo:FeCoNiO_x_/Mo:BiVO₄ photoanode delivered a record‐breaking photocurrent density of 7.15 mA cm⁻^2^ at 1.23 V_RHE_ (Figure S10, Supporting Information), surpassing not only its FeCoNiO_x_/Mo:BiVO₄ counterpart but also most state‐of‐the‐art BiVO₄‐based photoanodes reported to date (Figure 2f and Table S3, Supporting Information). Crucially, the transient photocurrent responses under chopped illumination fully aligned with steady‐state measurements, confirming the robustness of the observed performance enhancements.

To evaluate the solar‐to‐hydrogen conversion efficiency under applied bias, we calculated the applied bias photon‐to‐current efficiency (ABPE). As shown in Figure 2b, the Mo:FeCoNiO_x_/Mo:BiVO₄ photoanode achieves a remarkable ABPE of 2.56% at 0.66 V_RHE_, representing a sevenfold improvement over pristine BiVO₄ (0.35% at 0.89 V_RHE_). Further verification of photoconversion efficiency was obtained through incident photon‐to‐current efficiency (IPCE) measurements. The Mo:FeCoNiO_x_/Mo:BiVO₄ system demonstrates exceptional IPCE values of ≈96% in the 360–425 nm wavelength range (Figure 2c), significantly outperforming BiVO₄ (35%), Mo:BiVO₄ (53%), and FeCoNiO_x_/Mo:BiVO₄ (75%) photoanodes. Integrated photocurrent densities calculated from IPCE spectra (Figure S11, Supporting Information) show excellent agreement with the current densities obtained from J‐V measurements. In addition, stability tests reveal that both BiVO₄ and Mo:BiVO₄ town‐center photoanodes suffer severe photocurrent degradation, losing 82% and 83% of their initial current density within 2 h (Figure 2d). While FeCoNiO_x_ cocatalyst deposition provides moderate stability enhancement, the FeCoNiO_x_/Mo:BiVO₄ system retains only 14% of its initial performance after 20 h. In striking contrast, the Mo:FeCoNiO_x_/Mo:BiVO₄ photoanode maintains exceptional stability throughout extended 20‐h operation. Additionally, we employed gas chromatography (GC) to quantify the amounts of oxygen and hydrogen by a PEC water splitting cell that utilized Mo:FeCoNiO_x_/Mo:BiVO₄ as the photoanode and calculated the corresponding Faradaic efficiency (Figure S12, Supporting Information). Notably, the average Faradaic efficiency of oxygen production reached an impressive value of 97.7%, thereby indicating that the generated photocurrent originated from water oxidation reaction. As shown in Figure 2e,f, the comprehensive results demonstrate the superior performance of Mo:FeCoNiO_x_/Mo:BiVO₄ photoanode.^[^ ^45^, ^46^, ^47^, ^48^, ^49^, ^50^, ^51^, ^52^, ^53^, ^54^ ^]^ This further confirms that dual Mo doping significantly enhances its water oxidation kinetics.

### 2.3 Charge Transfer Behavior and Interfacial Kinetics

**Figure 3:**
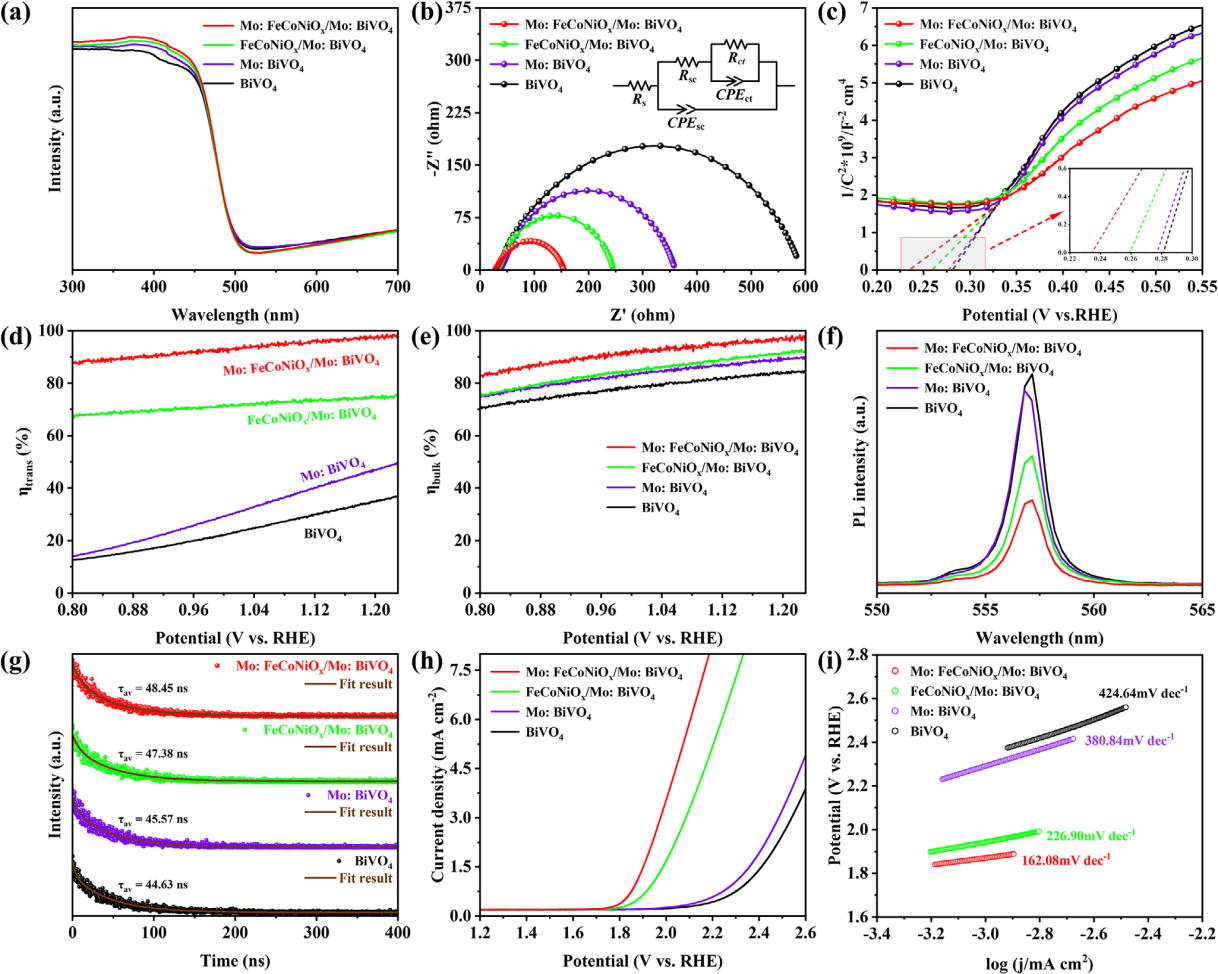
a) UV–vis spectra, b) EIS curves, c) Mott−Schottky plots measured under dark conditions, d) The interfacial hole transfer efficiency (η_trans_), e) The bulk charge separation efficiency (η_bulk_) f) PL spectra, g) Time‐resolved photoluminescence decay curves, h) LSV curves in the dark, and i) Tafel slope curves of BiVO₄, Mo:BiVO₄, FeCoNiO_x_/Mo:BiVO₄ and Mo:FeCoNiO_x_/Mo:BiVO₄ photoanodes.

The superior PEC performance of Mo:FeCoNiO_x_/Mo:BiVO₄ photoanodes originates from optimized charge transfer and interfacial kinetics, as revealed through comprehensive characterization. While ultraviolet visible spectroscopy (UV–vis) absorption spectra (**Figure** **3**a) show unchanged light absorption, electrochemical impedance spectroscopy (EIS) analysis (Figure 3b and Table S1, Supporting Information) demonstrates significantly reduced bulk (R_sc_ = 30.4 Ω vs 198.9 Ω for BiVO₄) and charge transfer resistances (R_ct_ = 100. Ω vs 358.4 Ω for BiVO₄), confirming enhanced charge transport.^[^ ^55^ ^]^ Mott–Schottky analysis reveals n‐type behavior with doubled donor density (11.37×10^2^⁰ vs 5.75×10^2^⁰ cm⁻^3^) and more negative flat‐band potential in the modified photoanode (Figure 3c and Table S2, Supporting Information). Open–circuit potential (OCP) decay curve exhibits a substantial enhanced ΔV_OC_ (0.267 V vs 0.218 V for BiVO₄), further validating its accelerated surface carriers transfer ability (Figure S13, Supporting Information).^[^ ^56^, ^57^ ^]^ Efficiency measurements show exceptional interfacial hole transfer (η_trans_ = 98.8%) and bulk charge separation (η_bulk_ = 96.5%) at 1.23 V_RHE_ (Figure 3d,e), supported by strongly quenched photoluminescence (PL) emission and increased average carrier lifetime (τ_av_) indicating suppressed recombination (Figure 3f,g). Moreover, the transient photocurrent curves (Figure S14, Supporting Information) revealed a pronounced attenuation of the spike signals, further supporting its effective suppression of surface carrier recombination. Combining the valence band edge potentials derived from Valence Band X‐ray Photoelectron Spectroscopy (VB‐XPS) and the band gap values derived from the UV−vis absorption spectra, we obtained the band structure of all samples (Figure S15c, Supporting Information). It is found that the conduction band (CB) and valence band (VB) of Mo:FeCoNiO_x_/Mo:BiVO₄ shifted to lower energy levels. This structure is more conducive to photogenerated holes drifting to the semiconductor‐electrolyte interface and electrons drifting to the back contact, thereby enhancing the charge transfer efficiency. Dark OER tests demonstrate superior catalytic activity with 260 mV lower onset potential and reduced Tafel slope (162.08 vs 424.64 mV dec⁻¹ for BiVO₄), confirming enhanced water oxidation kinetics (Figure 3h,i). Electrochemically active surface area (ECSA) evaluation shows a substantial increased ECSA value, further corroborating the enhanced water oxidation kinetics from the perspective of the active surface area (Figure S16e, Supporting Information).^[^ ^58^ ^]^ These results establish that dual Mo‐doping optimizes bulk charge transport and surface reaction kinetics.

### 2.4 Theoretical Study of the Mechanism

**Figure 4:**
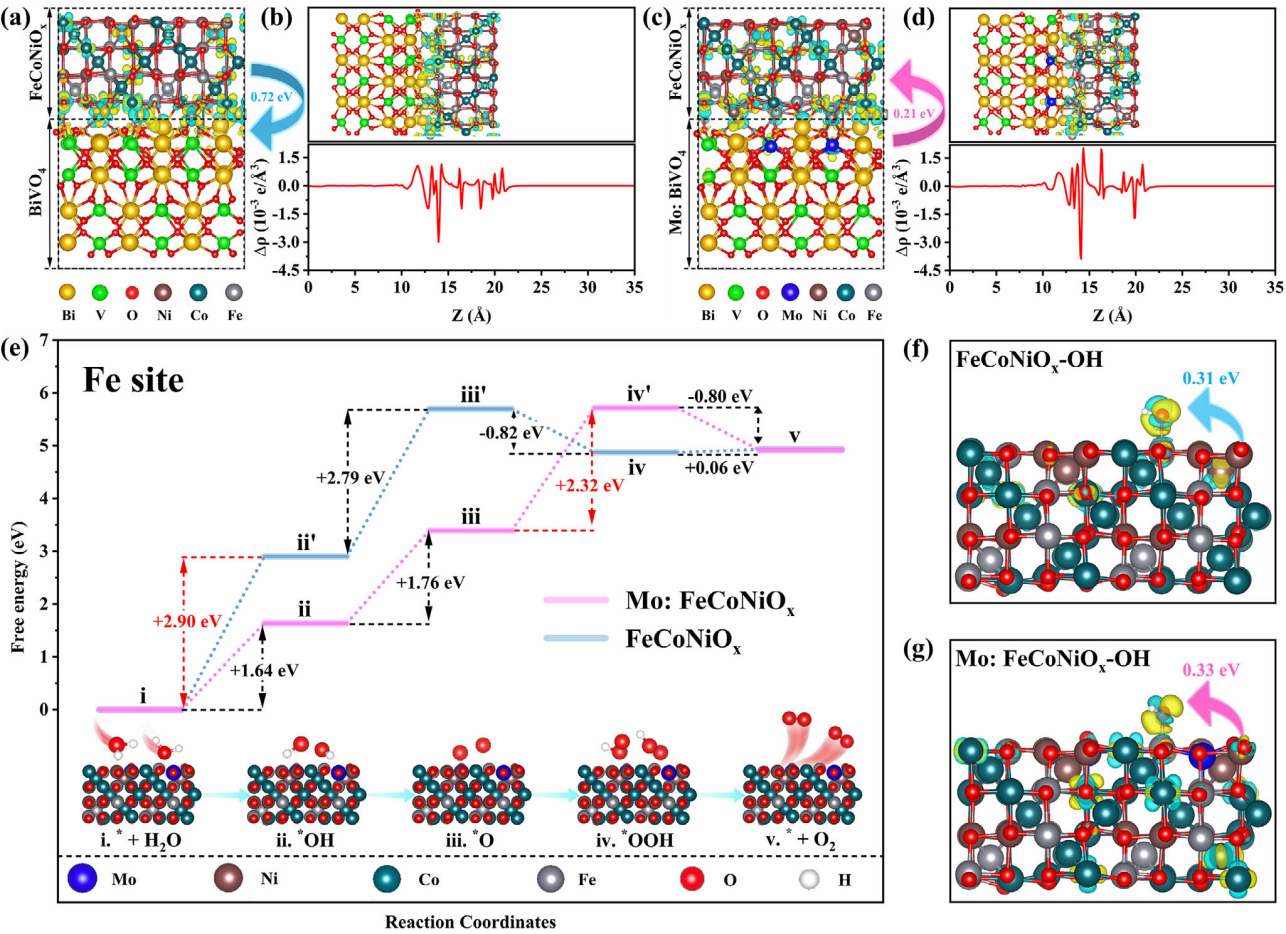
The Charge density difference mapping for a) FeCoNiO_x_/BiVO₄, c) FeCoNiO_x_/Mo:BiVO₄. The Planar‐averaged electron density difference Δρ(z) for b) FeCoNiO_x_/BiVO₄ and d) FeCoNiO_x_/Mo:BiVO₄. The yellow and cyan areas indicate electron accumulation and depletion, respectively. The isosurface of charge density is set to 0.0085 e Å^−3^. e) The Gibbs free energy profiles of OER reaction on FeCoNiO_x_ and Mo:FeCoNiO_x_. The calculated charge density differences of ^*^OH intermediate on FeCoNiO_x_ f) and Mo:FeCoNiO_x_ g).

Density functional theory (DFT) calculations elucidate the dual role of Mo doping in enhancing interfacial charge transfer and oxygen evolution reaction (OER) activity for the Mo:FeCoNiO_x_/Mo:BiVO₄ photoanode. Formation energy calculations (Figures S17 and S18, Supporting Information) confirm preferential Mo substitution at Ni (−8.24 eV) and V (−2.95 eV) sites in FeCoNiO_x_ and BiVO₄, respectively. Charge density difference analysis (**Figure** **4**a‐d) reveals localized interfacial charge redistribution, with Bader charge quantification showing 0.72 e^−^ transferred from FeCoNiO_x_ to BiVO₄ at their interface, while Mo doping in BiVO₄ reverses this direction (0.21 e^−^ from Mo:BiVO₄ to FeCoNiO_x_). This inversion facilitates hole accumulation in FeCoNiO_x_ under illumination, rationalizing the superior experimental OER performance of FeCoNiO_x_/Mo:BiVO₄ over FeCoNiO_x_/BiVO₄ (Figure S19, Supporting Information). Free energy profiling (Figure 4e and Figure S20, Supporting Information) identifies ^*^OH adsorption as the rate‐determining step, where Mo doping reduces the energy barrier at Fe sites from 2.90 eV (pristine FeCoNiO_x_) to 1.64 eV, outperforming Co/Ni sites (3.58/3.28 eV, Figure S21, Supporting Information). Charge density differences during ^*^OH adsorption (Figure 4f,g) and Bader analysis demonstrate enhanced electron transfer to ^*^OH intermediates (0.31 e^−^ → 0.33 e^−^) on Mo:FeCoNiO_x_, enhancing adsorption strength (Figure S22, Supporting Information) and accelerating OER kinetics. These results establish that Mo doping simultaneously optimizes bulk charge separation through interfacial electron redistribution and activates surface catalytic sites via electronic modulation, providing a unified mechanism for enhanced photoelectrochemical water splitting.

## 3 Conclusion

In summary, we demonstrate that dual electronic modulation through strategic Mo doping in both bulk BiVO₄ and surface FeCoNiO_x_ cocatalyst synergistically addresses the intrinsic limitations of BiVO₄ photoanodes for photoelectrochemical water splitting. By introducing Mo at V sites in BiVO₄ (bulk doping) and Ni sites in FeCoNiO_x_ (surface modification), optimized Mo:FeCoNiO_x_/Mo:BiVO₄ system achieves a near‐theoretical photocurrent density of 7.15 mA cm⁻^2^ (95.3% of the 7.50 mA cm⁻^2^ limit) at 1.23 V_RHE_ under AM 1.5 G illumination. The breakthrough performance stems from cross‐scale electronic reconstruction: Bulk Mo doping enhances n‐type conductivity and charge transport, while surface Mo incorporation redistributes electrons to create localized reservoirs at Fe/Co/Ni sites, simultaneously improving charge separation efficiency (96.5%, vs 84.9% for pristine BiVO₄) and accelerating hole migration kinetics (2.7‐fold). Combined DFT and experimental analyses reveal that Mo‐induced electronic reconfiguration enables dual‐functional Fe sites, acting as efficient hole‐trapping centers and highly active OER catalysts (ΔG_*OH_ reduced by 1.26 eV). This study offers a practical approach to designing high‐efficiency photoanodes and provides valuable insights into multi‐scale electronic engineering for advanced solar energy conversion.

## 4 Experimental Section

### Synthesis of Porous BiVO₄ Photoanodes

The preparation of the BiVO₄ photoanode was based on a previous report by Kim et al.^[^ ^26^ ^]^ Typically, 3.32 g KI was dissolved in 50 mL of deionized water and adjusted the pH to 1.7 with HNO₃. Then, 0.97 g Bi(NO₃)₃·5H₂O was added to the solution until it turned into an orange‐red transparent solution. Subsequently, 20 mL of ethanol solution containing 0.547 g p‐benzoquinone was mixed with the previous solution and stirred vigorously for 10 min for the subsequent electrodeposition. The BiOI (1×1 cm^2^) was performed at −0.1 V versus Ag/AgCl for 300s at room temperature using a CHI760E electrochemical workstation, FTO served as the working electrode (WE), Pt foil as the counter electrode (CE), and Ag/AgCl (4 m KCl) as the reference electrode (RE), respectively. Following this, the BiOI film was directly converted into the BiVO₄ photoanode. 160 µL DMSO solution containing 0.2 m VO(acac)₂ was dropped on the BiOI film and annealed in air at 450 °C for 2 h with a ramping rate of 2 °C min^−1^ to convert BiOI into BiVO₄ photoanode. After that, the annealed photoanode was immersed in 1.0 m KOH for 15 min to remove the excess V₂O₅ on the surface. Finally, the photoanode was rinsed with deionized water and dried in air to obtain the pure BiVO₄ photoanode.

### Synthesis of Mo:BiVO₄ Photoanodes

0.040 m H₃BO₃ and 0.020 m KOH were dissolved in 40 mL H₂O with stirring for 15 min to form the potassium borate (K₃BO₃, KBi) buffer solution. Then, 0.034 mm (NH₄)₆Mo₇O₂₄⋅4H₂O was added to the previous solution and stirred vigorously for 15 min. Using the above‐mentioned three‐electrode system, where the working electrode was the BiVO₄ photoanode. The system was operated for 20 min under 1 standard solar irradiation (100 mW cm^−^^2^) at a constant current density of −10 µA cm^−^^2^ to prepare the Mo:BiVO₄ photoanode. Finally, the photoanode was rinsed with deionized water and dried in air to obtain the pure Mo:BiVO₄ photoanode. According to the synthesis time (10, 20, 30 min) during the synthesis process, we denoted the Mo:BiVO₄ electrodes as 1‐Mo:BiVO₄, 2‐Mo:BiVO₄, and 3‐Mo:BiVO₄, respectively.

### Synthesis of FeCoNiO_x_/Mo:BiVO₄ Photoanodes

0.020 mm Co(NO₃)₂·6H₂O, 0.080 mm NiSO₄⋅6H₂O and 0.032 mm FeSO₄⋅7H₂O were added sequentially to 40 mL KBi buffer solution and stirred vigorously for 15 min. Using the above‐mentioned three‐electrode system, where the working electrode was the Mo:BiVO₄ photoanode. The FeCoNiO_x_ catalyst was deposited on the Mo:BiVO₄ surface under 1 standard solar irradiation (100 mW cm^−^^2^) at a constant current of −10 µA cm^−2^ for 420 s.

### Synthesis of Mo:FeCoNiO_x_/Mo:BiVO₄ Photoanodes

0.034 mm (NH₄)₆Mo₇O₂₄⋅4H₂O was added to 40 mL KBi buffer solution and stirred vigorously for 15 min. Using the above‐mentioned three‐electrode system, while the working electrode was the FeCoNiO_x_/Mo:BiVO₄ photoanode. The system was operated for 30 min under 1 standard solar irradiation (100 mW cm^−^^2^) at a constant electrical voltage of 0.472 V versus Ag/AgCl to prepare the Mo:FeCoNiO_x_/Mo:BiVO₄ photoanode. Finally, the photoanode was rinsed with deionized water and dried in air to obtain the pure Mo:FeCoNiO_x_/Mo:BiVO₄ photoanode. According to the synthesis time (15, 30, 45 min) during the synthesis process, we denoted the Mo:FeCoNiO_x_/Mo:BiVO₄ electrodes as 1‐Mo:FeCoNiO_x_/Mo:BiVO₄, 2‐Mo:FeCoNiO_x_/Mo:BiVO₄, and 3‐Mo:FeCoNiO_x_/Mo:BiVO₄, respectively.

## Conflict of Interest

The authors declare no conflict of interest.

## Supporting information



Supporting Information

## Data Availability

The data that support the findings of this study are available in the supplementary information of this article.
