# Supplementary material for: Dual Mo‐Doping in BiVO4/FeCoNiOx Photoanode Enables Near‐Theoretical Photocurrent Density via Synergistic Bulk‐Surface Engineering for Solar Water Splitting
Source: Adv Sci (Weinh). 2025 Jul 16;12(39):e09037. doi: 10.1002/advs.202509037 (PMC12533305; doi:10.1002/advs.202509037)
Supplement: Supplementary file 1 — Supporting Information [file ADVS-12-e09037-s001.docx]

**Supporting Information**

**Dual Mo-Doping in BiVO₄/FeCoNiO_x_ Photoanodes Enables Near-Theoretical Photocurrent Density via Synergistic Bulk-Surface Engineering for Solar Water Splitting**

Rongzhe Zhao, Yuchen Zhou, Peng Guo, Rong Mo, Yonghua Tang,* and Hongxing Li*

**Note S1. Chemicals and Materials**

All chemicals were procured and utilized as received, without any additional purification. Bismuth nitrate pentahydrate (Bi(NO₃)₃⋅5H₂O, 99 %, Shanghai Macklin reagent medicament company), potassium iodide (KI, AR, Tianjin Kermel chemical reagent company), nitric acid (HNO₃, AR, Hunan Huihong reagent company), p-benzoquinone (C₆H₄O₂, 97%, Shanghai Macklin reagent medicament company), absolute ethanol(CH₃CH₂OH, AR, Hunan Huihong reagent company), vanadyl acetylacetonate (VO(acac)₂, 99%, Shanghai Macklin reagent medicament company), dimethyl sulfoxide (DMSO, 99.9%, Sigma-Aldrich reagent medicament company), potassium hydroxide (KOH, AR, Shantou Xihua reagent company), boric acid (H₃BO₃, AR, Shanghai Hushi reagent company), iron sulfate heptahydrate (FeSO₄⋅7H₂O, 99%, Shanghai Aladdin reagent company), cobalt nitrate hexahydrate (Co(NO₃)₂·6H₂O, 99%, Shanghai Macklin reagent medicament company), nickel sulfate hexahydrate (NiSO₄⋅6H₂O, 98.5%, Shanghai Macklin reagent medicament company), ammonium molybdate tetrahydrate ((NH₄)₆Mo₇O₂₄⋅4H₂O, ≥81%, Shanghai Macklin reagent medicament company), sodium sulfite (Na₂SO₃, AR, Xilong chemical reagent company), fluorine-doped tin oxide (FTO) substrates (1cm×1.8 cm, Dalian Heptachroma company).

**Note S2. Photoelectrochemical measurements**

The PEC performances of all the fabricated photoanodes were tested using a three-electrode system on an electrochemical analyzer (CHI760E). The samples, Pt foil (2 cm × 2 cm), and saturated Ag/AgCl electrode were used as working, counter, and reference electrodes, respectively. 0.25 mol/L K₃BO₃ buffer (pH = 9.5) was employed as the electrolyte. A xenon lamp source (Microsolar300 Beijing Per­fect light) was used as light source to generate the simulated AM 1.5G illumination. The light irradiation direction in all tests was back side. The incident light illumination intensity was calibrated to 100 mW cm⁻² with a digital power meter before tests. The potentials vs. Ag/AgCl electrode were converted to the potential vs. RHE using the Nernst equation:

$$E_{RHE}=E_{Ag/AgCl}+0.059\times pH+E_{Ag/AgCl}^{0}$$

Where *E_RHE_* refers to the converted potential vs. RHE, pH represents the pH value of electrolyte, E_Ag/AgCl_ denotes the measured potential vs. Ag/AgCl, and $E_{Ag/AgCl}^{0}$= 0.197 at 25 ℃. The chopped LSV curves were generated at a scan rate of 5 mV s⁻¹ and a time interval of 5 s to switch the light illumination in a potential range of 0 V_RHE_ ~ 1.23 V_RHE_.

Applied bias photon-to-current efficiency (ABPE) can be calculated using the following equation:

Where *J_ph_* is the photocurrent density obtained under an applied bias (*V_bias_*), and *P_total_* is the incident illumination power density.

The Incident-photon-to-current conversion efficiency (IPCE) was measured at 1.23 V_RHE_ and calculated using the following equation:

$$IPCE\left(\%\right)=\frac{1240\times J\left(mA/cm^{2}\right)}{\lambda\left(nm\right)\times P_{light}\left(mW/cm^{2}\right)}\times100$$

Where *J* is the measured photocurrent density at each specific wavelength, λ is the wavelength of the incident light and *P_light_* is the light power density at a specific wavelength.

The estimated photocurrent densities (*J_c_*) were calculated by integrating the IPCE values with the standard solar spectrum (ASTMG-173-03) using the following equation:

$$J_{c}=\int_{320}^{600} \frac{\lambda\times IPCE\left(\lambda\right)\times E\left(\lambda\right)}{1240}d\left(\lambda\right)$$

Specifically, λ and *E*(λ) represent the light wavelength (nm) and the corresponding power density (mW cm⁻²).

For EIS measurements, a sinusoidal voltage pulse of 10mV amplitude was applied on a bias potential, with frequencies that ranged from 100 kHz to 0.1Hz.

The evolution of H_2_ and O_2_ was performed in a 0.25 M K_3_BO_3_ electrolyte at 1.23 V_RHE_ under AM 1.5 G illumination (100 mWcm^−2^) by an online gas analysis system (GC 7900 gas analyzer) equipped with a fully enveloped chamber (C108-2, Gaoss Union). The Faradaic efficiency (FE) was calculated using the follow equation:

$$FE_{H_{2}}=\frac{2n_{H_{2}}\times F}{\int_{0}^{t(s)} J(A)dt}\times100\%$$

$$FE_{O_{2}}=\frac{4n_{O_{2}}\times F}{\int_{0}^{t(s)} J(A)dt}\times100\%$$

where *F* (mol C^−1^) is Faraday’s constant, $n_{H_{2}}$(mol) is the mole number of hydrogen gas produced, $n_{O_{2}}$(mol) is the mole number of oxygen gas produced, *I* (A cm^−2^) is the photocurrent generated at a fixed bias of 1.23 V_RHE_ and *t* is the illumination time (s) under solar simulated AM1.5G light irradiation.

The determination of the surface charge transfer efficiencies ($\eta_{trans}$) can be achieved through the utilization of the subsequent equations:

$$\eta_{trans}=\frac{J_{H_{2}O}}{J_{Na_{2}SO_{3}}}$$

The symbols $J_{H_{2}O}$ and $J_{Na_{2}SO_{3}}$ denote the photocurrent densities acquired in 0.25 M potassium borate electrolytes with a pH of 9.5, in the absence and presence of 0.2 M Na₂SO₃, respectively.

The determination of the bulk charge separation efficiencies ($\eta_{bulk}$) can be achieved through the utilization of the subsequent equations:

$$\eta_{bulk}=\frac{J_{Na_{2}SO_{3}}}{J_{abs}}$$

The symbol *J_abs_* was the theoretical current density assuming the complete conversion of the absorbed irradiation. And all photoanodes performed nearly the same UV-Vis spectra with the non-doped BiVO₄ (Figure S8), so the *J_abs_* of all photoanodes was approximated to 7.5 mA cm⁻² based on the previous report.

Mott–Schottky plots were tested at a frequency of 600 Hz under dark condition, using the Mott–Schottky equation:

$$1/C^{2}=\frac{\left[\left(E-E_{fb}\right)-\left(k_{B}T/e\right)\right]\times2}{\left(\varepsilon\varepsilon_{0}eN_{d}\right)}$$

C is the space charge capacitance in the semiconductor, E is the applied potential, E*_fb_* is the flat band potential, *k_B_* is the Boltzmann constant, *T* is absolute temperature, *e* is permittivity of vacuum, *ε* is relative permittivity (ε=68) for BiVO₄, *ε*_0_ is vacuum permittivity, *N_d_* is the donor density.

Meanwhile, the donor density can be calculated with the transformed equation:

$$N_{d}=\left(\frac{2}{e\varepsilon\varepsilon_{0}}\right)\left[\frac{d\left(1/C^{2}\right)}{dV}\right]^{-1}$$

Where $\left[\frac{d\left(1/C^{2}\right)}{dV}\right]$ was the straight slope.

The time-resolved photoluminescence decay curves can be analyzed by biexponential function fitting, and the average recombination life­ time (τ_av_) was calculated by the following equation:

$$R(t)=A_{1}e^{(-t/\tau_{1})}+A_{2}e^{(-t/\tau_{2})}$$

$$\tau_{\text{av}}=\frac{A_{1}\tau_{1}^{2}+A_{2}\tau_{2}^{2}}{A_{1}\tau_{1}+A_{2}\tau_{2}}$$

In the formula, τ_1_ and τ_2_ are defined as the decay time for the fluorescence intensity, representing the speed of carrier recombination.

**Note S3. Materials characterization**

The morphologies of various BiVO₄ films were observed using a field emission scanning electron microscope (SEM, Zeiss Sigma 300). Microstructure analyses of the BiVO₄ films were carried out via trans­mission electron microscopy (TEM, JEOL JEM-F200). Phase analysis of the products was performed via XRD with Cu Kα radiation (λ = 1.5405 Å). Raman spectra were monitored on a laser confocal Raman spec­trometer (Renishaw inVia InSpect) with an excitation wavelength of 532 nm. The UV–vis absorption spectra were recorded on an Ideaoptics spectrophotometer (Shimadzu UV-3600). The bandgap values of the samples were calculated using the Tauc plot method:

$$(\alpha h\upsilon)^{1/n}=A(h\upsilon-E_{g})$$

where *α* means the absorbance, *h* is the Planck constant, *ν* represents the optical frequency, the value of *n* is 1/2 owing to the indirect bandgap semiconductor of the photoanodes, *A* is a constant relating to material properties, and *E_g_* is the bandgap energy. XPS measurements were used to analyze the surface compositions and binding energies of BiVO₄ films (Thermo Scientific K-Alpha). All binding energies in XPS spectra were referenced to the C 1s peak at 284.8 eV from the adventitious carbon. PL spectra were recorded by Wetic alpha 300 R with 532 nm laser excitation at room temperature.

**Note S4. DFT calculations**

All density functional theory (DFT) calculations were carried out using the CP2K software package (version 2024.1)^[1]^. For metal atoms, the wave functions were expanded using the molecularly optimized double-zeta Gaussian basis set (DZVP-MOLOPT-SR-GTH), while for non-metal atoms, the TZV2P-MOLOPT-GTH basis set was employed. An auxiliary plane-wave basis set was utilized with a cutoff energy of 420 Ry and a relative cutoff energy (rel_cutoff energy) of 55 Ry. To describe the weak interactions in the system, the DFT-D3 van der Waals correction method proposed by Grimme was applied^[2]^. Brillouin zone integration was performed using a reciprocal space mesh consisting solely of the gamma point. For bismuth vanadate (BiVO₄), we adopted its most stable 001 surface, with the surface unit cell structure comprising 36 bismuth (Bi) atoms, 36 vanadium (V) atoms, and 144 oxygen (O) atoms. The FeCoNiO_x_ layer structure contained 48 cobalt (Co), 24 nickel (Ni), and 24 iron (Fe) atoms. Geometry optimizations were conducted using the wavelet Poisson solver in conjunction with the Broyden−Fletcher−Goldfarb−Shanno (BFGS) algorithm until the DFT energy convergence criterion reached 5 × 10⁻⁶ Hartree, and the force acting on each atom was below 0.00045 Hartree/Bohr. All input file preparations and post-processing tasks were facilitated by the Multiwfn software package^[3-4]^. The Gibbs free energy changes (ΔG) for all reaction steps were calculated using the standard hydrogen electrode (SHE) method^[5]^, as described by the following equation:

G = E + ZPE − TS

where E, ZPE, and S represent the electronic free energy, zero-point energy, and entropy of the model at T=298.15 K, respectively.

The adsorption energy (E*_ads_*) was calculated using the following formula:

E*_ads_* = E*_total_* − (E*_surface_* + E*_adsorbate_*)

where:

E*_total_*: The total energy of the system after adsorption (the combined energy of the surface and the adsorbed molecule).

E*_surface_*: The total energy of the surface before adsorption.

E*_adsorbate_*: The total energy of the adsorbed molecule.


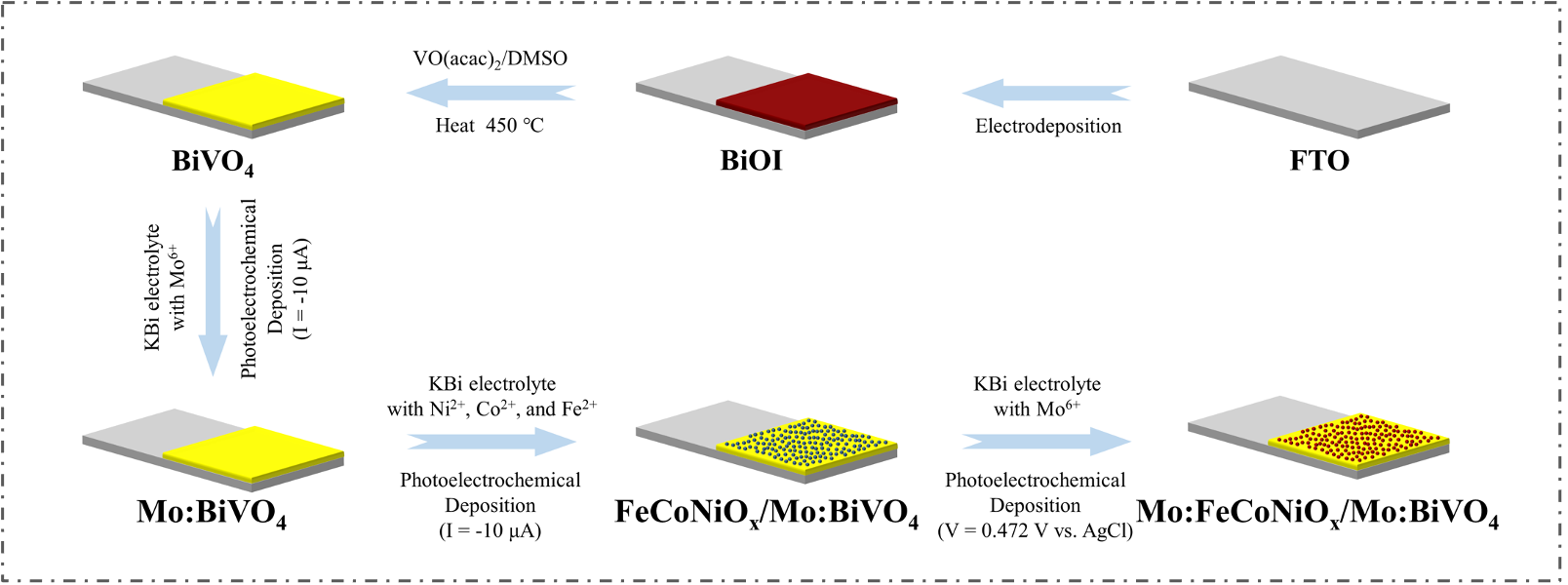


**Figure S1.** The synthesis diagram of Mo:FeCoNiO_x_/Mo:BiVO₄ photoanode.


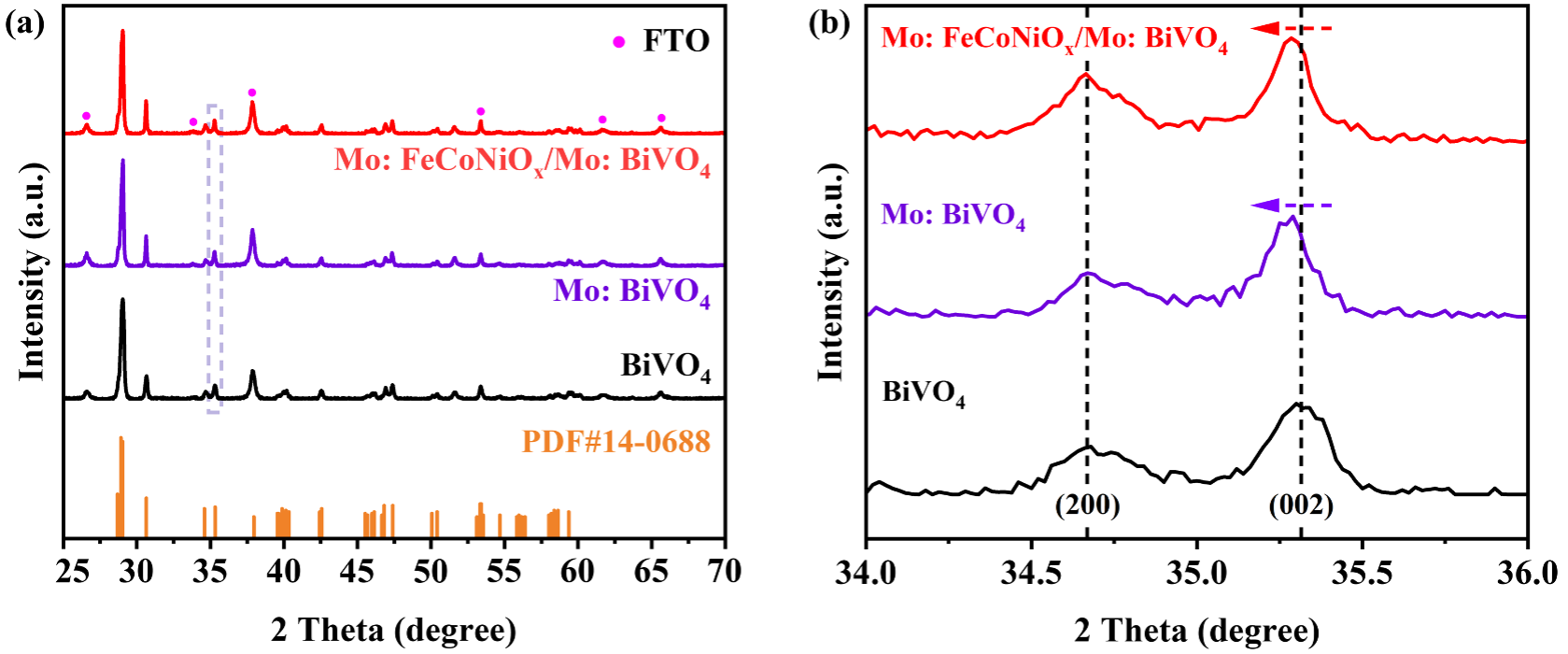


**Figure S2.** a) XRD patterns of BiVO₄, Mo:BiVO₄, and Mo:FeCoNiO_x_/Mo:BiVO₄ photoanodes. b) Partial enlargement of the selective area in (a).


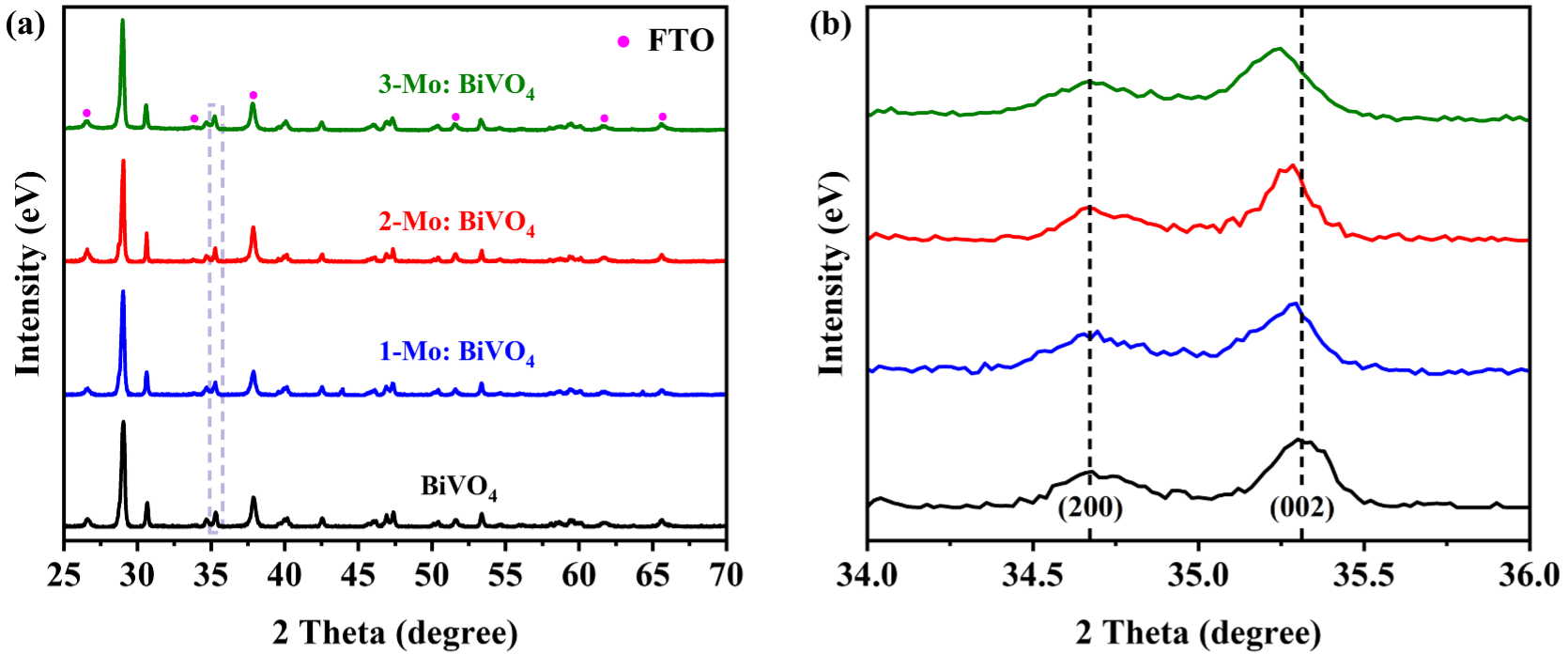


**Figure S3.** a) XRD patterns of BiVO₄ photoanode with different Mo doping content. b) Partial enlargement of the selective area in (a).


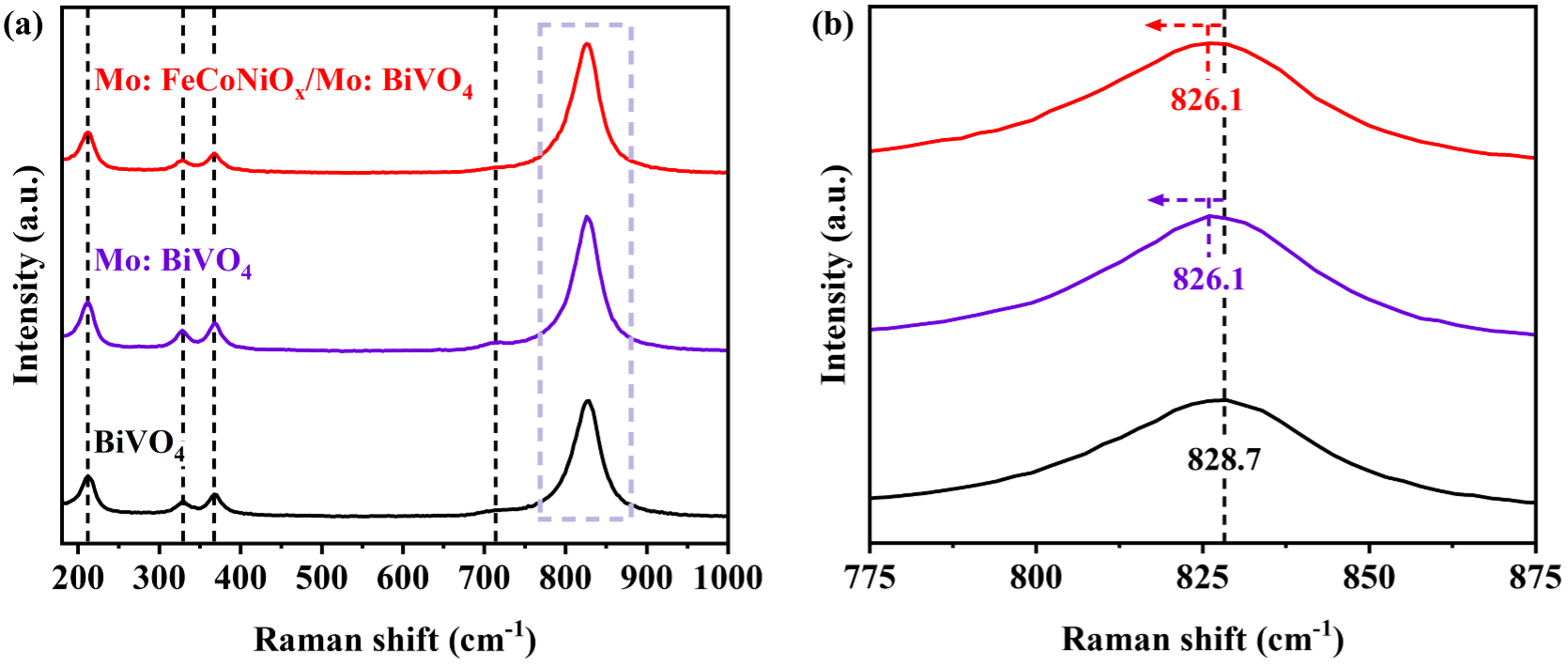


**Figure S4.** a) Raman spectra of BiVO₄, Mo:BiVO₄, and Mo:FeCoNiO_x_/Mo:BiVO₄ photoanodes. b) Partial enlargement of the selective area in (a).


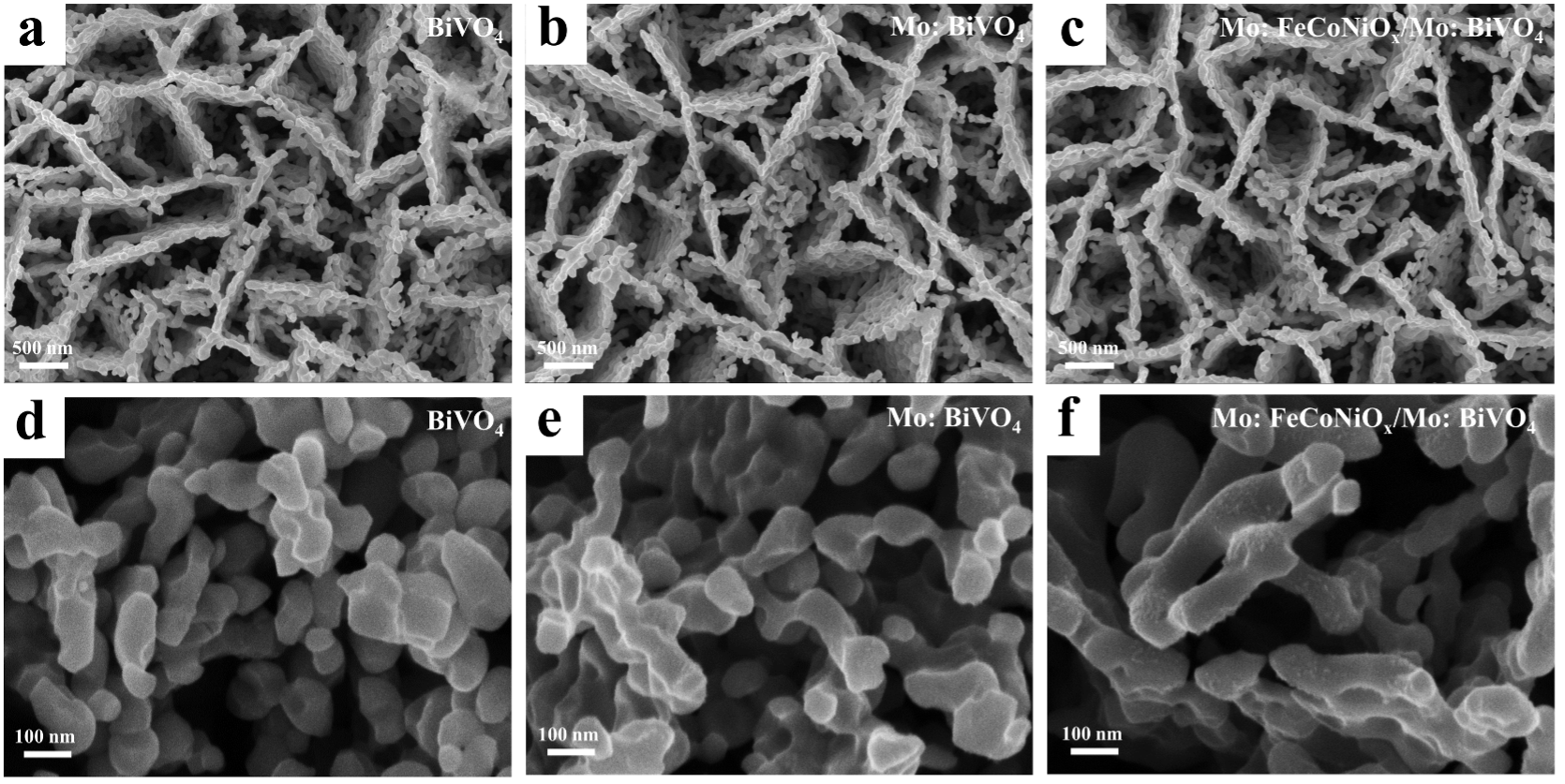


**Figure S5.** SEM images of BiVO₄, Mo:BiVO₄, and Mo:FeCoNiO_x_/Mo:BiVO₄ photoanodes.


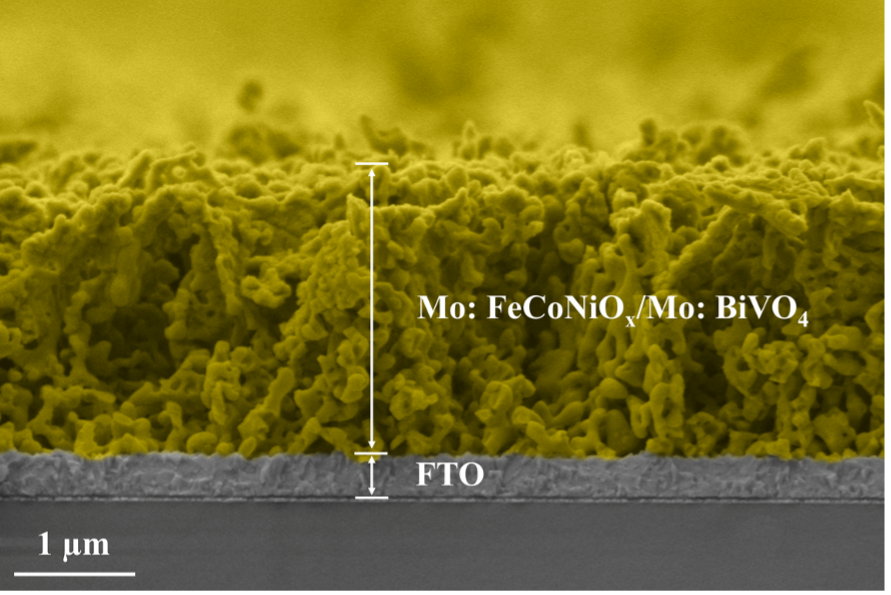


**Figure S6.** Cross sectional SEM image of Mo:FeCoNiO_x_/Mo:BiVO₄ photoanode.


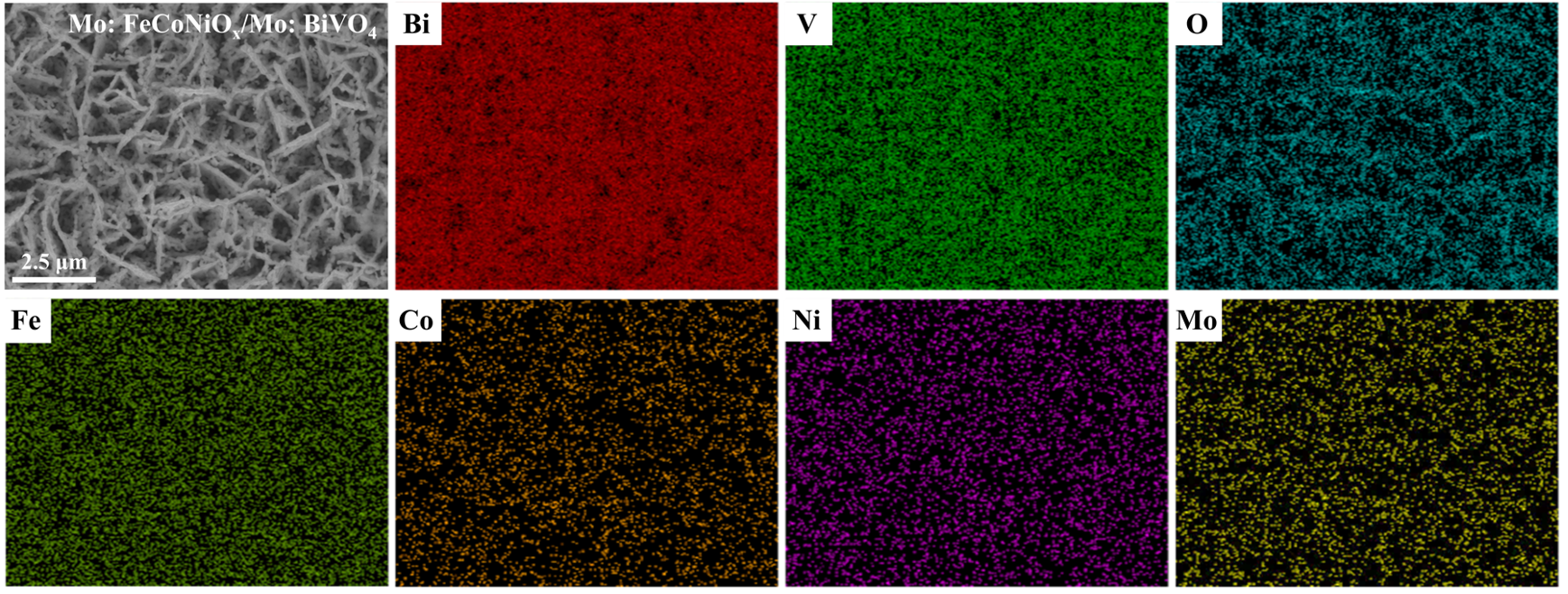


**Figure S7.** The SEM-EDS element mapping analysis of Mo:FeCoNiO_x_/Mo:BiVO₄ photoanode.


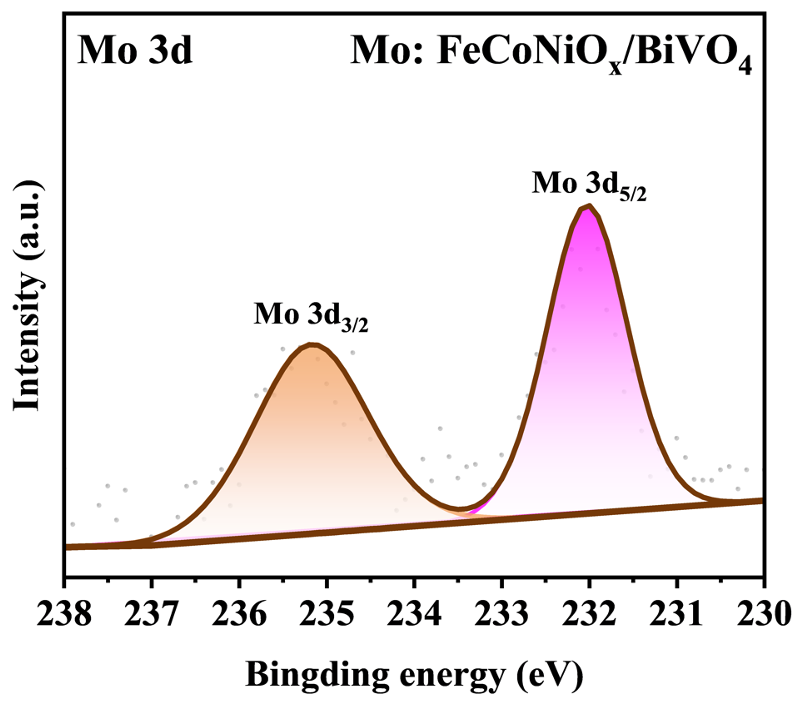


**Figure S8.** Mo 3d spectra of Mo:FeCoNiO_x_/BiVO₄ photoanode.


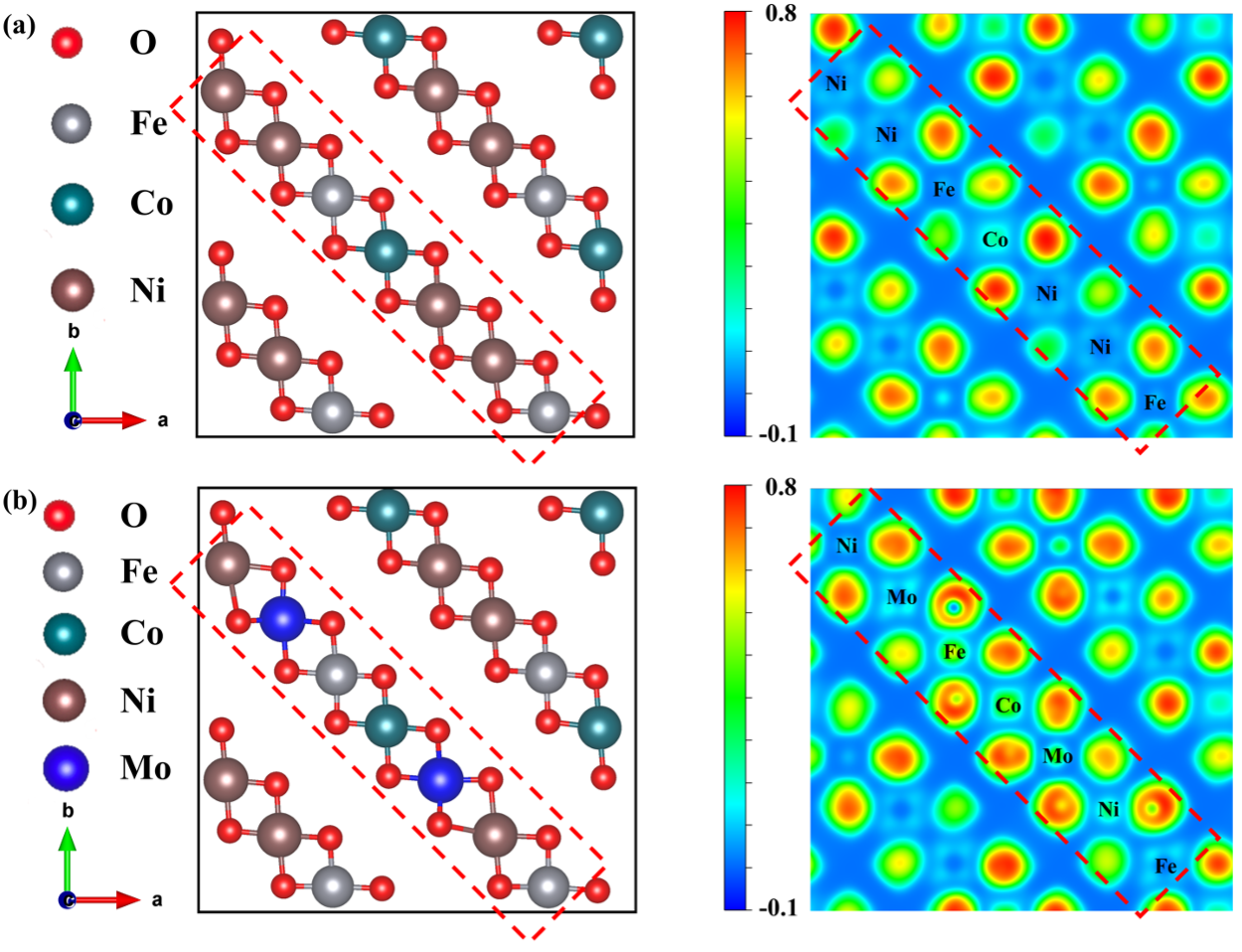


**Figure S9.** Electron localization functions (ELFs) of specific cross-sections in (a) FeCoNiO_x_ and (b) Mo:FeCoNiO_x_ cocatalyst.


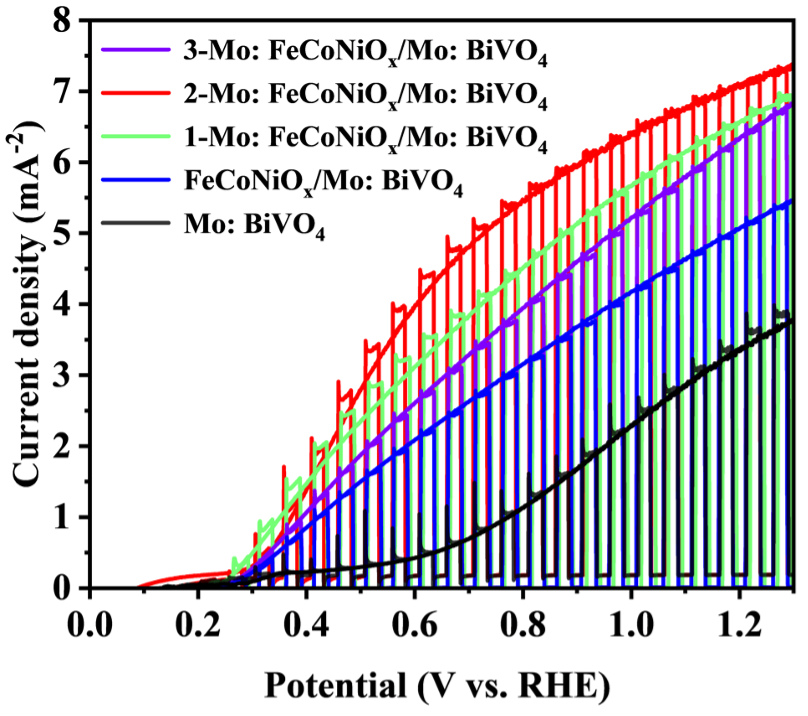


**Figure S10.** J-V curves of Mo: BiVO₄ and FeCoNiO_x_/Mo: BiVO₄ with different Mo doping content.


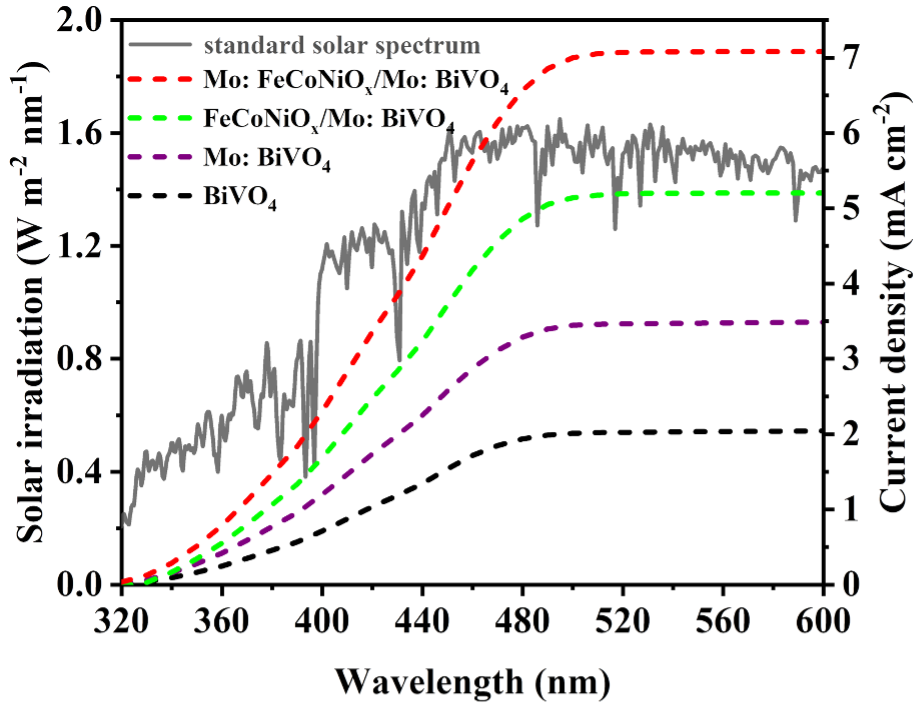


**Figure S11.** Calculated photocurrent densities by integrating corresponding IPCE curves (Fig. 2c) in over the photon flux of AM 1.5G illumination.


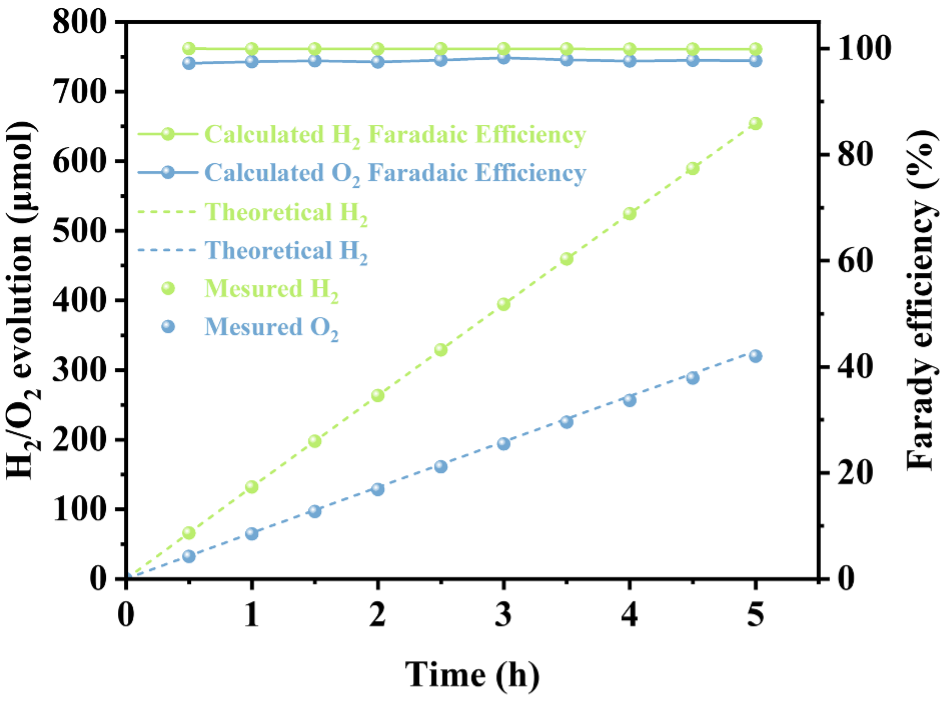


**Figure S12.** H_2_ and O_2_ evolution and faradaic efficiencies of Mo:FeCoNiO_x_/Mo:BiVO₄ photoanode.


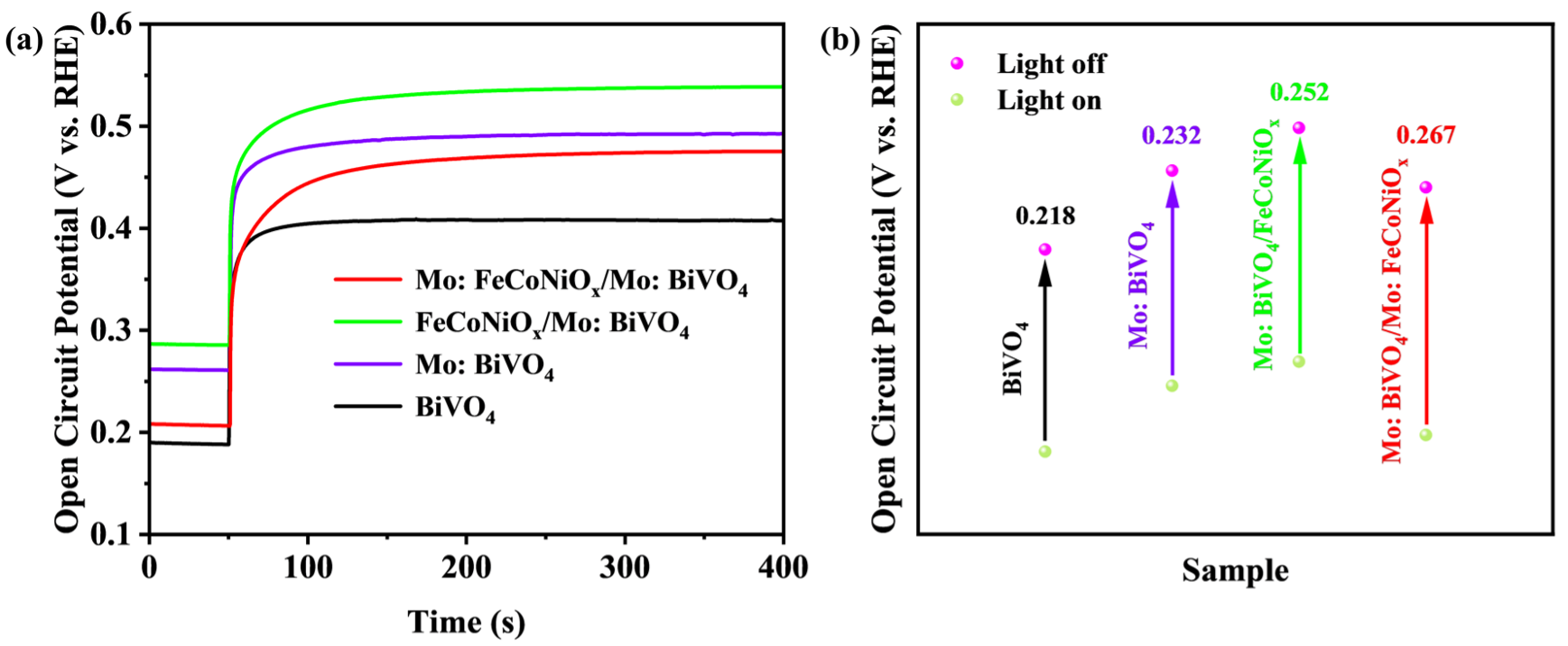


**Figure S13.** (a) OCP decay curves and (b) ΔVOC of BiVO₄, Mo:BiVO₄, FeCoNiO_x_/Mo:BiVO₄ and Mo:FeCoNiO_x_/Mo:BiVO₄ photoanodes.


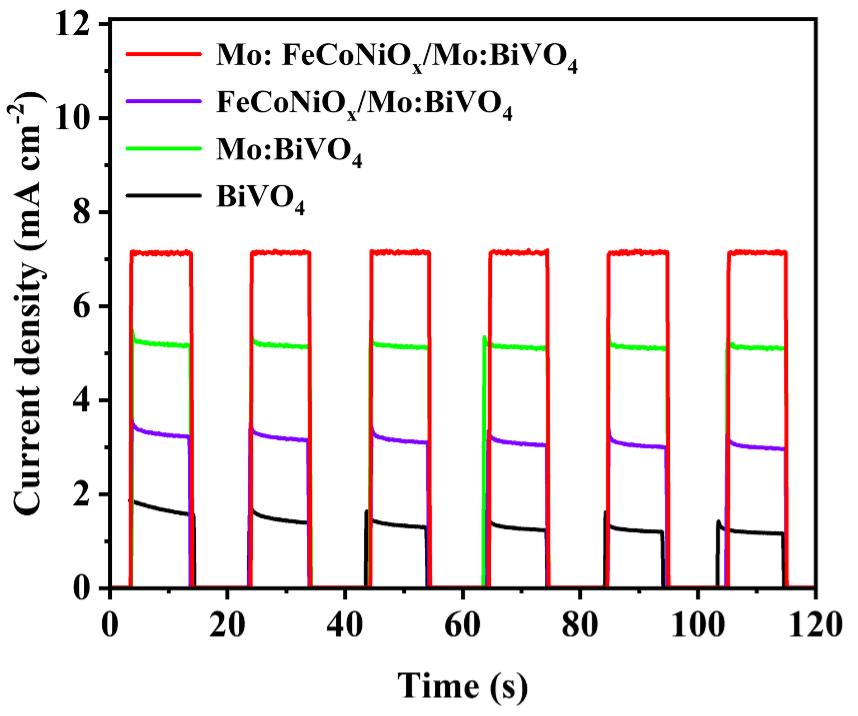


**Figure S14.** Transient photocurrent curves of BiVO₄, Mo:BiVO₄, FeCoNiO_x_/Mo:BiVO₄, and Mo:FeCoNiO_x_/Mo:BiVO₄ photoanodes.

**
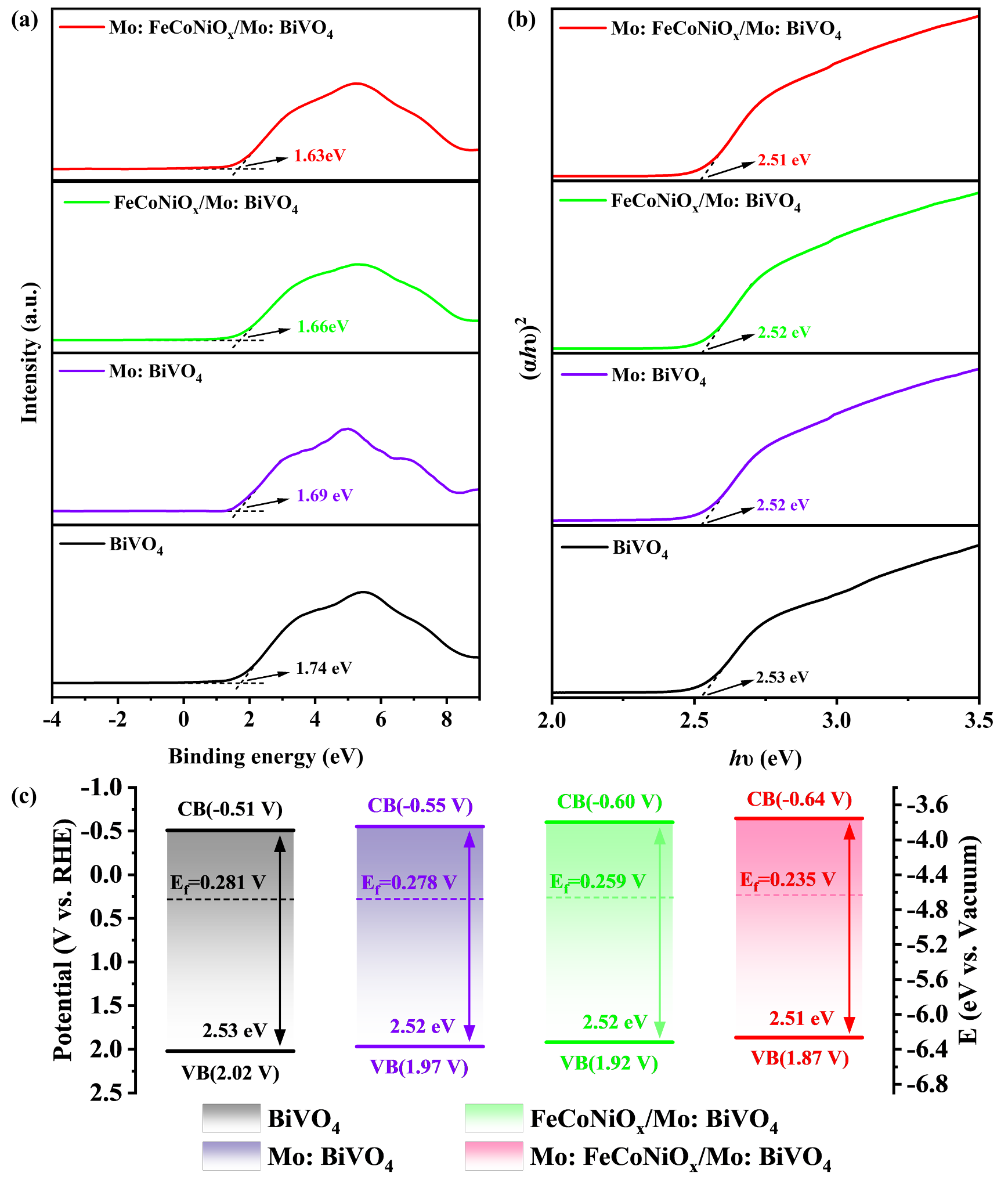
**

**Figure S15.** (a) VB-XPS curves, (b) The transformed Kubelka–Munk function versus the energy spectra, and (c) schematic illustration of band structure of BiVO₄, Mo:BiVO₄, FeCoNiO_x_/Mo:BiVO₄, and Mo:FeCoNiO_x_/Mo:BiVO₄ photoanodes.


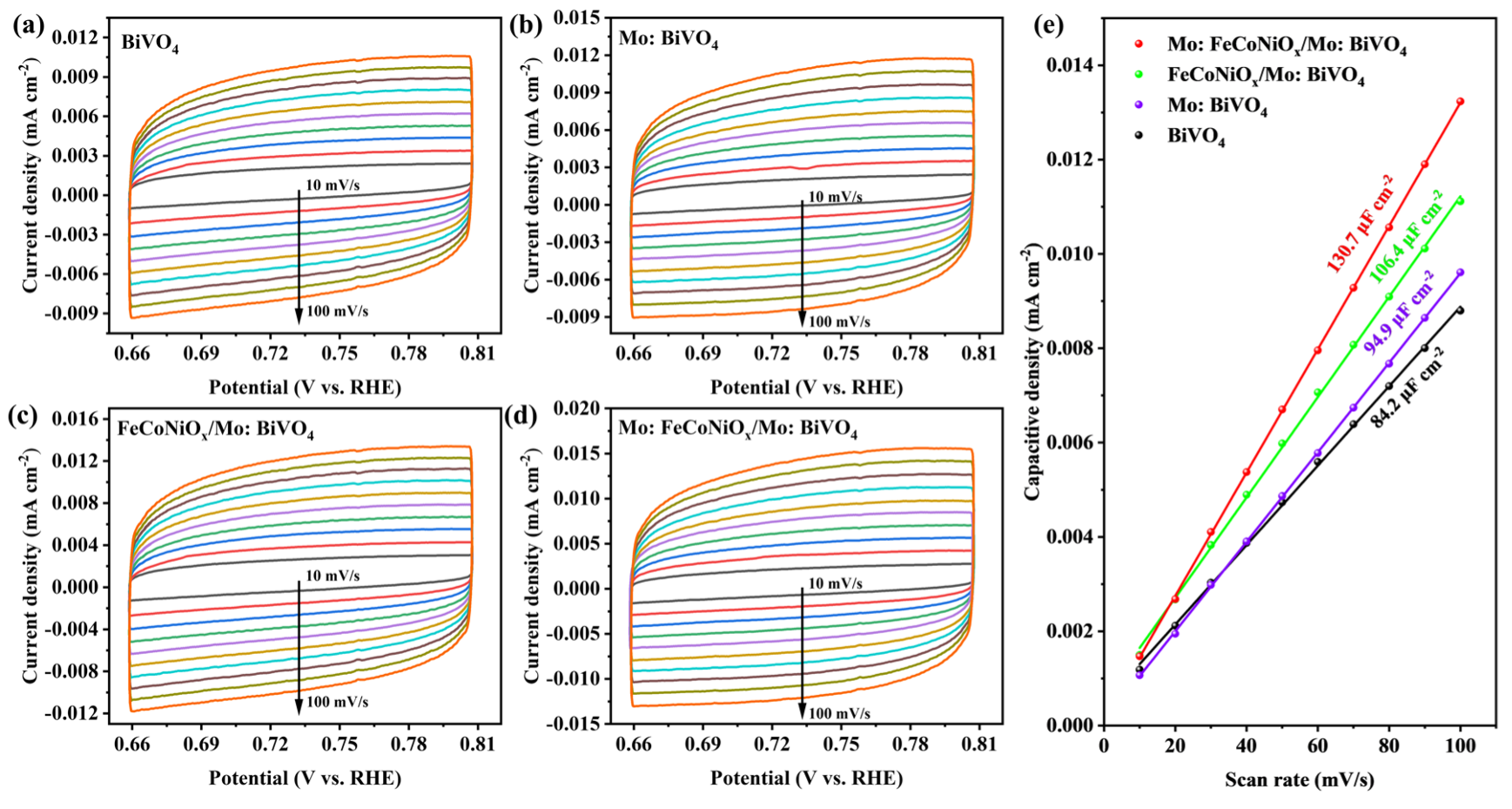


**Figure S16.** CV curves of (a) BiVO₄, (b) Mo:BiVO₄, (c) FeCoNiO_x_/Mo:BiVO₄, and (d) Mo:FeCoNiO_x_/Mo:BiVO₄. (e) ECSA evaluation of the obtained photoanodes.


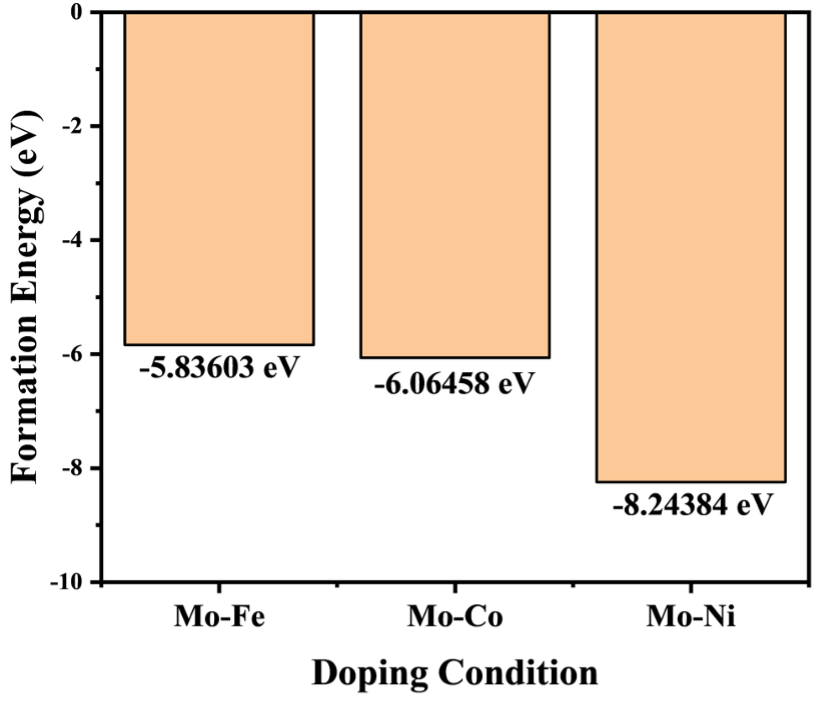


**Figure S17.** The formation energies of different molybdenum doping conditions in Mo:FeCoNiO_x_ cocatalyst.


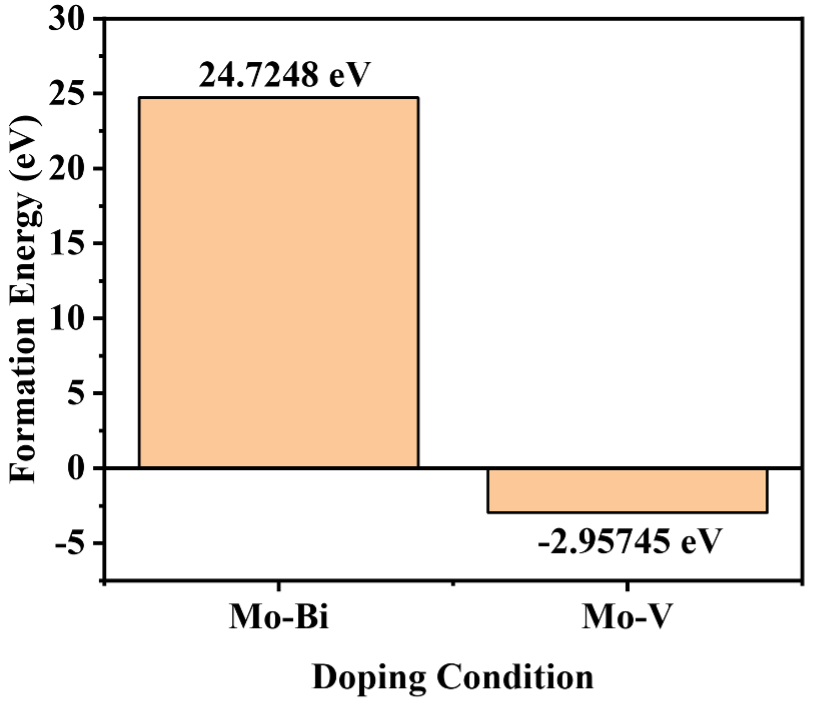


**Figure S18.** The formation energies of different molybdenum doping conditions in Mo:BiVO₄ photoanode.


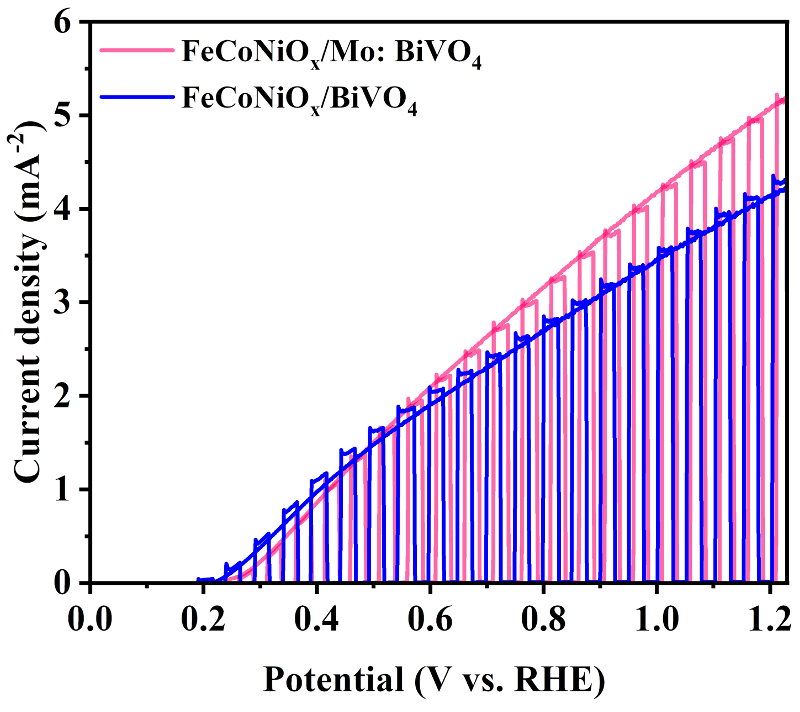


**Figure S19.** J-V curves of FeCoNiO_x_/Mo:BiVO₄ and FeCoNiO_x_/BiVO₄ photoanodes.


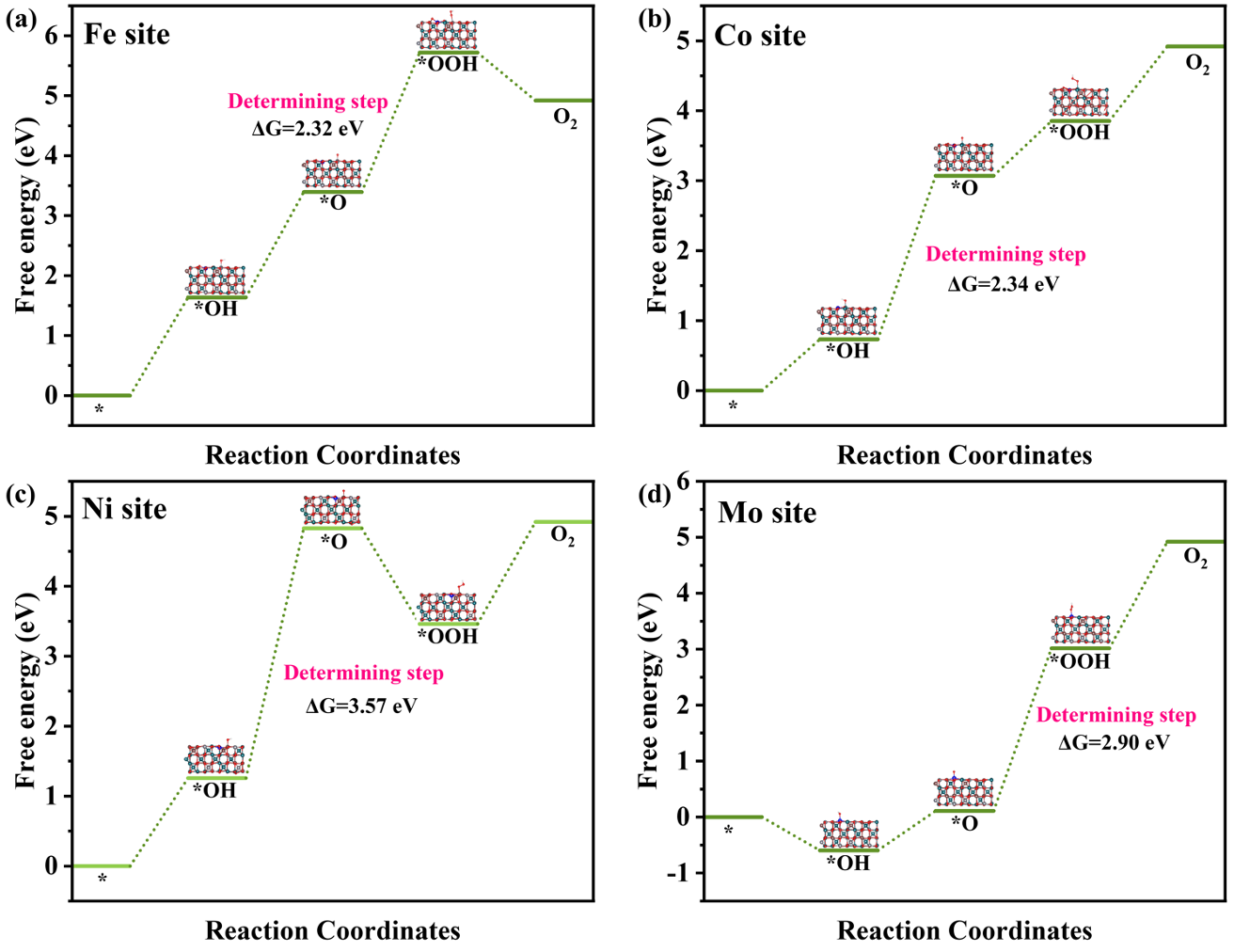


**Figure S20.** Gibbs free energy changes of OER for (a) Fe site, (b) Co site, (c) Ni site, and (d) Mo site on Mo:FeCoNiO_x_ cocatalyst.


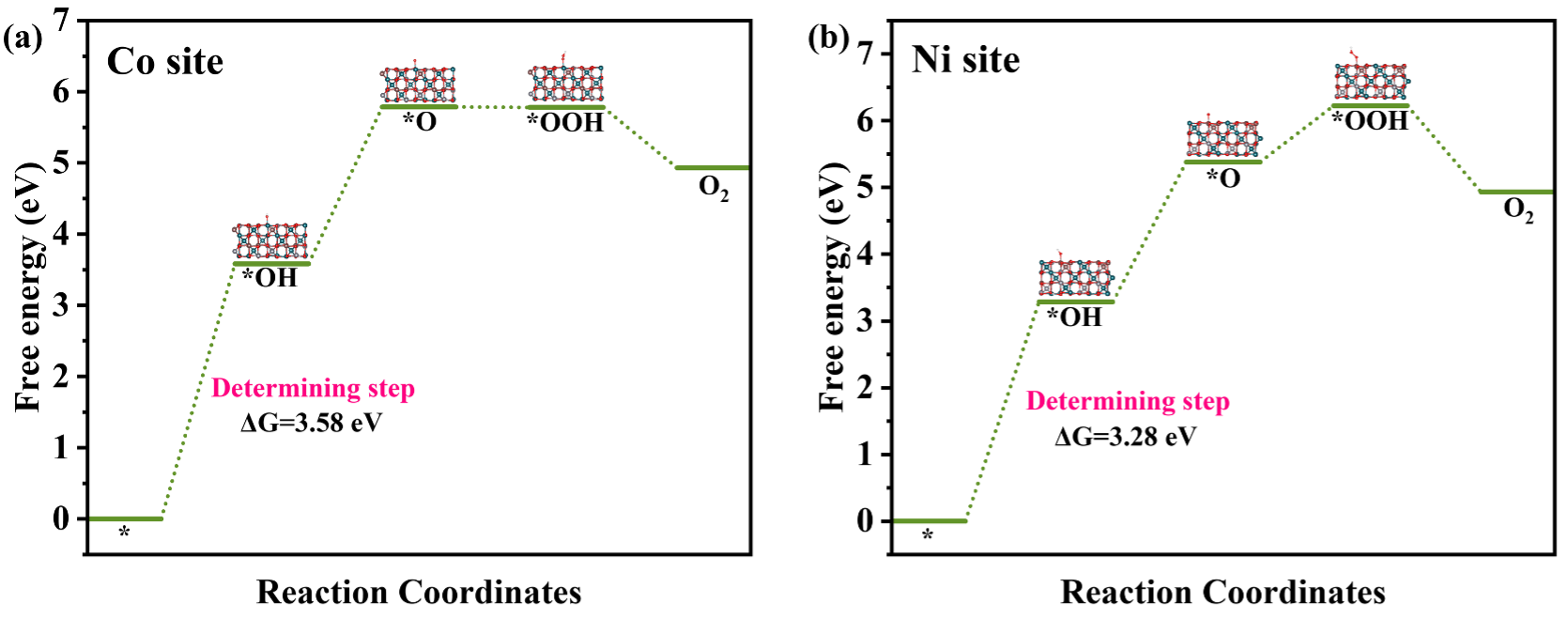


**Figure S21.** Gibbs free energy changes of OER for (a) Co site and (b) Ni site on FeCoNiO_x_ cocatalyst.


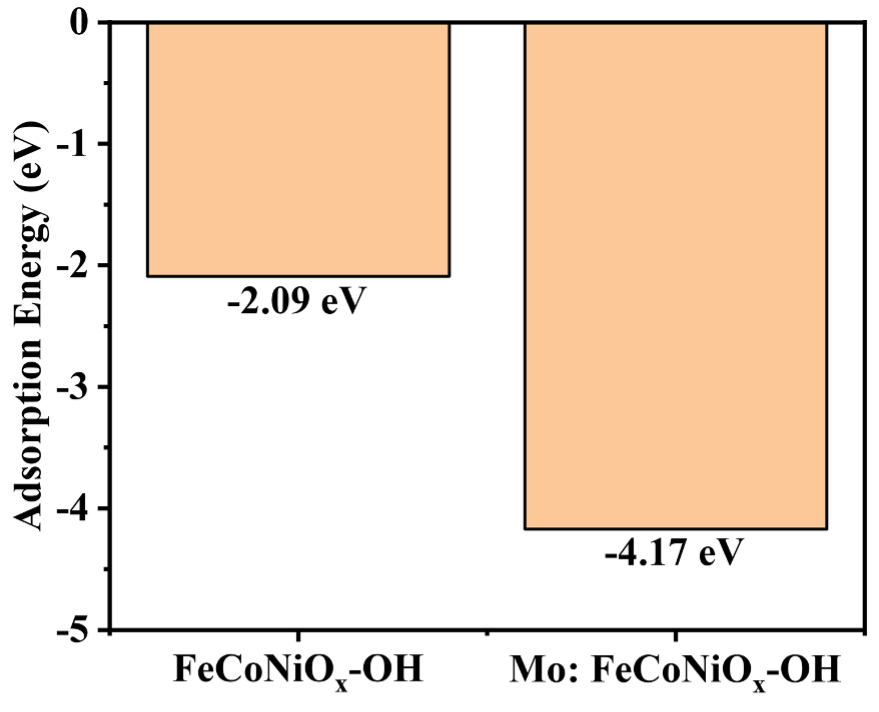


**Figure S22.** The adsorption energies of *OH intermediate on FeCoNiO_x_ and Mo:FeCoNiO_x_ cocatalysts.

**Table S1** The fitted R_s_, R_sc_, CPE_sc_, R_ct_, CPE_ct_, and chi-squared (*Χ*^2^) values of the photoanodes from EIS spectra.

| **Photoanode** | **R_s_**  **[Ω cm²]** | **R_sc_**  **[Ω cm]** | **CPE_sc_**  **[F cm^-2^]** | **R_ct_**  **[Ω cm]** | **CPE_ct_**  **[F cm^-2^]** | ***Χ*^2^**  **[10^-3^]** |
| --- | --- | --- | --- | --- | --- | --- |
| **BiVO₄** | **35.78** | **198.9** | **4.34E-5** | **358.4** | **1.48E-5** | **4.74** |
| **Mo:BiVO₄** | **37.91** | **67.78** | **9.20E-5** | **254.2** | **4.46E-5** | **8.46** |
| **Mo:FeCoNiO_x_/BiVO₄** | **32.25** | **23.85** | **1.21E-4** | **190.0** | **1.96E-5** | **7.35** |
| **Mo:FeCoNiO_x_/Mo:BiVO₄** | **28.40** | **30.36** | **3.04E-4** | **100.2** | **8.22E-5** | **9.18** |

**Table S2** The flat band potential (E_fb_) and carrier densities (N_d_).

| **Photoanode** | **E_fb_/V** | **N_d_/10²⁰ cm^-^³** |
| --- | --- | --- |
| **BiVO₄** | **0.281** | **5.753** |
| **Mo:BiVO₄** | **0.278** | **6.206** |
| **FeCoNiO_x_/Mo:BiVO₄** | **0.259** | **7.945** |
| **Mo:FeCoNiO_x_/Mo:BiVO₄** | **0.235** | **11.369** |

**Table S3** Comparison of our photoanode to other BiVO₄-based photoanodes.

| **Photoanode** | **Photocurrent**  **(1.23 V_RHE_)** | **ABPE**  **(max)** | **Stability** | **Ref** |
| --- | --- | --- | --- | --- |
| BiVO₄/FeNiPO_x_ | 6.73 mA/cm² | - | 84h | [6] |
| Mo:BiVO₄/CPE-TCB/NiFeCoO_x_ | 6.66 mA/cm² | - | 5h | [7] |
| BiVO₄/N:NiFeO_x_ | 6.4 mA/cm² | 1.90% | 5h | [8] |
| BiVO₄/Co_3_O₄/NiOOH | 6.4 mA/cm² | 2.69% | 90h | [9] |
| BiVO₄/Fe_x_Ni_1-x_OOH | 5.8 mA/cm² | 1.52% | 3h | [10] |
| BiVO₄/FeCoNiO_x_/Mo-MnO_y_ | 6.18 mA/cm² | 1.97% | 30h | [11] |
| BiVO₄/MQD/MoO_x_/NiFeOOH | 5.85 mA/cm² | 2.43% | 100h | [12] |
| BiVO₄/NiFeY LDH | 5.2 mA/cm² | - | 25h | [13] |
| BiVO₄/FeOOH/TANi | 4.6 mA/cm² | 1.30% | 3h | [14] |
| BiVO₄/NiFe-MoFs | 4.61 mA/cm² | 1.81% | 3h | [15] |
| BiVO₄/FeCoO_x_ | 4.82 mA/cm² | 1.16% | 10h | [16] |
| BiVO₄/β-FeOOH | 4.3 mA/cm² | 0.71% | 2h | [17] |
| BiVO₄/FeOOH/CQDs | 2.53 mA/cm² | 0.60% | 2h | [18] |
| BiVO₄/VOx | 6.29 mA/cm² | 2.37% | 40h | [19] |
| BiVO₄/Vo-FeNiOOH | 3.76 mA/cm² | 1.07% | 10h | [20] |
| BiVO₄/Ni-N₄-O/NiOOH | 6.0 mA/cm² | 2.66% | 20h | [21] |
| BiVO₄/NiB | 6.0 mA/cm² | 2.03% | 30h | [22] |
| BiVO₄/CoNi-MoFs | 3.2 mA/cm² | 0.65% | 3h | [23] |
| BiVO₄/Co-Bi | 3.2 mA/cm² | 1.10% | 1h | [24] |
| Mo:BiVO₄/B-C₃N₄/NiFeO_x_ | 6.2 mA/cm² | 2.67% | 10h | [25] |
| Fe-N-BiVO₄/FeNiOOH | 7.01 mA/cm² | 1.28% | 20h | [26] |
| Mo:BiVO₄/Gr/CoPi | 4.36 mA/cm² | 1.42% | 20h | [27] |
| Mo:BiVO₄/H-Co-Ci | 3.15 mA/cm² | 0.73% | 3h | [28] |
| C-Mo:BiVO₄/NiFe | 5.62 mA/cm² | 1.89% | 6h | [29] |
| Mo:BiVO₄/FeCoO_x_ | 4.55 mA/cm² | 1.20% | 20h | [30] |
| Mo:BiVO₄/Fe:VOPO₄ | 6.59 mA/cm² | 1.96% | 10h | [31] |
| Mo:BiVO₄/Mo:FeCoNiO_x_ | 7.15mA/cm² | 2.56% | 20h | This work |

**References**

[1] J. Hutter, M. Iannuzzi, F. Schiffmann, J. VandeVondele, CP2K: atomistic simulations of condensed matter systems, *Wires. Comput. Mol. Sci.* **2014**, *4*, 15.

[2] S. Grimme, J. Antony, S. Ehrlich, H. Krieg, A consistent and accurate ab initio parametrization of density functional dispersion correction (DFT-D) for the 94 elements H-Pu, *J. Chem. Phys.* **2010**, *132*, 154104.

[3] T. Lu, A comprehensive electron wavefunction analysis toolbox for chemists, Multiwfn, *J. Chem. Phys.* **2024**, *161*, 082503.

[4] T. Lu, F. Chen, Multiwfn: a multifunctional wavefunction analyzer, *J. Comput. Chem.* **2012**, *33*, 580.

[5] J. K. Nørskov, J. Rossmeisl, A. Logadottir, L. Lindqvist, Origin of the Overpotential for Oxygen Reduction at a Fuel-Cell Cathode, *J. Phys. Chem. B.* **2004**, *108*, 17886.

[6] Z. Zhang, X. Huang, B. Zhang, Y. Bi, High-performance and stable BiVO₄ photoanodes for solar water splitting via phosphorus–oxygen bonded FeNi catalysts, *Energy Environ. Sci.* **2022**, *15*, 2867.

[7] J. W. Yang, S. G. Ji, C.-S. Jeong, J. Kim, H. R. Kwon, T. H. Lee, S. A. Lee, W. S. Cheon, S. Lee, H. Lee, M. S. Kwon, J. Moon, J. Y. Kim, H. W. Jang, High-efficiency unbiased water splitting with photoanodes harnessing polycarbazole hole transport layers, *Energy Environ. Sci.* **2024**, *17*, 2541.

[8] B. Zhang, S. Yu, Y. Dai, X. Huang, L. Chou, G. Dong, Y. Bi, Nitrogen-incorporation activates NiFeO_x_ catalysts for efficiently boosting oxygen evolution activity and stability of BiVO₄ photoanodes, *Nat. Commun.* **2021**, *12*, 6969.

[9] Y. Zhang, L. Xu, B. Liu, X. Wang, T. Wang, X. Xiao, S. Wang, W. Huang, Engineering BiVO₄ and Oxygen Evolution Cocatalyst Interfaces with Rapid Hole Extraction for Photoelectrochemical Water Splitting, *ACS Catal.* **2023**, *13*, 5938.

[10] B. Zhang, X. Huang, Y. Zhang, G. Lu, L. Chou, Y. Bi, Unveiling the Activity and Stability Origin of BiVO₄ Photoanodes with FeNi Oxyhydroxides for Oxygen Evolution, *Angew. Chem. Int. Ed.* **2020**, *59*, 18990.

[11] Y. Zhou, H. Li, P. Guo, Y. Zhang, P. Zhou, Simultaneous enhancement of charge transfer and surface catalysis through a polymetallic oxide cocatalyst on BiVO₄ photoanodes for highly efficient and stable water oxidation, *Chem. Eng. J.* **2024**, *489*, 151220.

[12] Y. Song, X. Zhang, Y. Zhang, P. Zhai, Z. Li, D. Jin, J. Cao, C. Wang, B. Zhang, J. Gao, L. Sun, J. Hou, Engineering MoO_x_/MXene Hole Transfer Layers for Unexpected Boosting of Photoelectrochemical Water Oxidation, *Angew. Chem. Int. Ed.* **2022**, *61*, e202200946.

[13] D. He, R.-T. Gao, S. Liu, M. Sun, X. Liu, K. Hu, Y. Su, L. Wang, Yttrium-Induced Regulation of Electron Density in NiFe Layered Double Hydroxides Yields Stable Solar Water Splitting, *ACS Catal.* **2020**, *10*, 10570.

[14] T. Tian, G. Jiang, Y. Li, W. Xiang, W. Fu, Unveiling the activity and stability of BiVO₄ photoanodes with cocatalyst for water oxidation, *Renew Energy.* **2022**, *199*, 132.

[15] Y. Li, Q. Wang, X. Hu, Y. Meng, H. She, L. Wang, J. Huang, G. Zhu, Constructing NiFe-metal-organic frameworks from NiFe-layered double hydroxide as a highly efficient cocatalyst for BiVO₄ photoanode PEC water splitting, *Chem. Eng. J.* **2022**, *433*, 133592.

[16] S. Wang, T. He, J.-H. Yun, Y. Hu, M. Xiao, A. Du, L. Wang, New Iron‐Cobalt Oxide Catalysts Promoting BiVO₄ Films for Photoelectrochemical Water Splitting, *Adv. Funct. Mater.* **2018**, *28*, 1802685.

[17] B. Zhang, L. Wang, Y. Zhang, Y. Ding, Y. Bi, Ultrathin FeOOH Nanolayers with Abundant Oxygen Vacancies on BiVO₄ Photoanodes for Efficient Water Oxidation, *Angew. Chem. Int. Ed.* **2018**, *57*, 2248.

[18] T. Zhou, S. Chen, J. Wang, Y. Zhang, J. Li, J. Bai, B. Zhou, Dramatically enhanced solar-driven water splitting of BiVO₄ photoanode via strengthening hole transfer and light harvesting by co-modification of CQDs and ultrathin β-FeOOH layers, *Chem. Eng. J.* **2021**, *403*, 126350.

[19] B. Liu, X. Wang, Y. Zhang, L. Xu, T. Wang, X. Xiao, S. Wang, L. Wang, W. Huang, A BiVO₄ Photoanode with a VO_x_ Layer Bearing Oxygen Vacancies Offers Improved Charge Transfer and Oxygen Evolution Kinetics in Photoelectrochemical Water Splitting, *Angew. Chem. Int. Ed.* **2023**, *62*, e202217346.

[20] R. Zhang, X. Ning, Z. Wang, H. Zhao, Y. He, Z. Han, P. Du, X. Lu, Significantly Promoting the Photogenerated Charge Separation by Introducing an Oxygen Vacancy Regulation Strategy on the FeNiOOH Co-Catalyst, *Small.* **2022**, *18*, 2107938.

[21] X. Zhang, P. Zhai, Y. Zhang, Y. Wu, C. Wang, L. Ran, J. Gao, Z. Li, B. Zhang, Z. Fan, L. Sun, J. Hou, Engineering Single-Atomic Ni-N₄-O Sites on Semiconductor Photoanodes for High-Performance Photoelectrochemical Water Splitting, *J. Am. Chem. Soc.* **2021**, *143*, 20657.

[22] R. Gao, N. T. Nguyen, T. Nakajima, J. He, X. Liu, X. Zhang, L. Wang, L. Wu, Dynamic semiconductor-electrolyte interface for sustainable solar water splitting over 600 hours under neutral conditions, *Sci. Adv.* **2023**, *9*, 4589.

[23] S. Zhou, K. Chen, J. Huang, L. Wang, M. Zhang, B. Bai, H. Liu, Q. Wang, Preparation of heterometallic CoNi-MOFs-modified BiVO₄: a steady photoanode for improved performance in photoelectrochemical water splitting, *App. Catal. B: Environ.* **2020**, *266*, 118513.

[24] S. Wang, P. Chen, J.-H. Yun, Y. Hu, L. Wang, An Electrochemically Treated BiVO₄ Photoanode for Efficient Photoelectrochemical Water Splitting, *Angew. Chem. Int. Ed.* **2017**, *56*, 8500.

[25] K. Ye, H. Li, D. Huang, S. Xiao, W. Qiu, M. Li, Y. Hu, W. Mai, H. Ji, S. Yang, Enhancing photoelectrochemical water splitting by combining work function tuning and heterojunction engineering, *Nat. Commun.* **2019**, *10*, 3687.

[26] J. Yang, C. Deng, Y. Lei, M. Duan, Y. Yang, X. Chen, S. Yang, J. Li, H. Sheng, W. Shi, C. Chen, J. Zhao, Fe-N Co-Doped BiVO₄ Photoanode with Record Photocurrent for Water Oxidation, *Angew. Chem. Int. Ed.* **2025**, *137*, e202416340.

[27] M. Fang, Q. Cai, Q. Qin, W. Hong, W. Liu, Mo-doping induced crystal orientation reconstruction and oxygen vacancy on BiVO₄ homojunction for enhanced solar-driven water splitting, *Chem. Eng. J.* **2021**, *421*, 127766.

[28] X. Hu, Q. Wang, Y. Li, Y. Meng, L. Wang, H. She, J. Huang, The hydrophilic treatment of a novel co-catalyst for greatly improving the solar water splitting performance over Mo-doped bismuth vanadate, *J. Colloid Interface Sci.* **2022**, *607*, 219.

[29] S. Zhang, T. Duan, D. Yan, H. Yang, L. Cheng, Y. Pei, K. You, H. a. Luo, Hole transfer layer of glucose-derived carbon enabled rapid charge transfer and hole storage for efficient water splitting, *Chem. Eng. J.* **2024**, *486*, 150176.

[30] K. Xue, L. Yu, C. Liu, H. Luo, Y. Zhang, H. Zhu, W. Xing, Rational tailoring of spin-polarized photoelectrode for magnetic-assisted overall water splitting, *Chem. Eng. J.* **2024**, *497*, 154474

[31] B. He, Y. Cao, K. Lin, M. Wu, Y. Zhu, X. Cui, L. Hu, Y. Yang, X. Liu, Enhanced bulk and interfacial charge transfer in Fe:VOPO₄ modified Mo:BiVO₄ photoanodes for photoelectrochemical water splitting, *eScience.* **2025**, *5*, 100242.
